# Vaginal host immune-microbiome-metabolite interactions associated with spontaneous preterm birth in a predominantly white cohort

**DOI:** 10.1038/s41522-025-00671-4

**Published:** 2025-03-26

**Authors:** Megan Cavanagh, Emmanuel Amabebe, Neha S. Kulkarni, Maria D. Papageorgiou, Heather Walker, Matthew D. Wyles, Dilly O. Anumba

**Affiliations:** 1 Division of Clinical Medicine, University of Sheffield, Sheffield, UK; 2 School of Biosciences, University of Sheffield, Sheffield, UK; 3 Sheffield Institute for Translational Neuroscience (SITraN), University of Sheffield, Sheffield, UK

**Keywords:** Microbiome, Biological techniques, Microbiology, Health care

## Abstract

In order to improve spontaneous preterm birth (sPTB) risk stratification in a predominantly white cohort of non-labouring pregnant women, we analysed their vaginal microbiota, metabolite, cytokine and foetal fibronectin (FFN) concentrations at two gestational time points (GTPs): GTP1 (20^+0^–22^+6^ weeks, preterm = 17; term = 32); and GTP2 (26^+0^–28^+6^ weeks, preterm = 14; term = 31). At GTP1, the preterm-delivered women showed abundant *G. vaginalis* (AUC = 0.77) over *L. crispatus* and *L. iners*, and upregulation of 10 metabolites. At GTP2, the same women had more lactobacilli- and mixed anaerobes-dominated microbiota, upregulation of five metabolites, and decreased TNFR1, distinguishing them from their term counterparts (AUC = 0.88). From GTP1 to GTP2, sPTB was associated with increased microbiota α-diversity, and upregulation of pantothenate and urate. CXCL10 declined in the term-delivered women by ~3-fold, but increased in the preterm-delivered women (AUC = 0.68), enhanced by FFN (AUC = 0.74). Characterising the complex dynamic interactions between cervicovaginal microbial metabolites and host immune responses could enhance sPTB risk stratification.

## Introduction

The cervicovaginal (CV) metabolome is a dynamic reflection of host-microbiota interactions^1–6^. Changes in the vaginal microbial-metabolite composition during pregnancy can trigger subclinical inflammatory responses that can activate the pathway to labour and lead to spontaneous preterm birth (sPTB), i.e. delivery <37 weeks gestation^7–15^. PTB is a global health burden and the major cause of death in children <5 years of age. PTB is a complex syndrome, with the majority (~75%) of the cases occurring spontaneously due to multiple aetiologies^16^.

Intrauterine infection and inflammation, commonly ascending from a dysbiotic vaginal microflora, account for ~50% of spontaneous PTB (sPTB)^16–18^. The vaginal environment, which is a unique ecosystem with interactions between vaginal epithelial cells, microorganisms and the host immune system^19^, is dominated (up to 99%) by *Lactobacillus* spp. with low bacterial diversity^1,20^. The vaginal microbiome and metabolome are dynamic in non-pregnant and pregnant states^1–6^. The temporal microbiota-induced metabolic activities can directly and indirectly initiate subclinical inflammatory responses characterised by dysregulated expression of cytokines and chemokines that recruit immune cells to gestational tissues, such as the decidua, chorioamnion and cervix as well as the foetus^14,21–23^. Such microbiota-induced inflammatory changes can occur before conception and during pregnancy^3,23,24^, and can disrupt foetal membranes leading to leakage of foetal fibronectin into the vaginal space^25–27^, which is associated with increased risk of sPTB^26,27^.

We have previously reported increased expression of pro-inflammatory RANTES and IL-1β, with an associated high prevalence of vaginal anaerobic species, from mid to late second trimester in women without symptoms of preterm labour (PTL) or clinical infection who eventually delivered preterm^11^. In symptomatic women without clinical infection, our previous work has also shown altered metabolite and cytokine profiles through mid-gestation (19–36 weeks)^10^. The current study sought to link these temporal changes in metabolite and cytokine profiles to changes in vaginal microbiota in a predominantly white cohort. We also sought to determine the metabolic pathways most likely involved in these phenotypes, in order to clarify the pathomechanism of inflammatory sPTB linked to vaginal dysbiosis.

Bacterial colonisation of the choriodecidual membranes and non-infectious stimuli (i.e., sterile inflammation) can trigger immunological responses from both the mother and foetus, leading to the production of inflammatory mediators that activate the common pathway to spontaneous labour^28–30^. Inflammatory cytokines produced in the female genital tract are a dynamic reflection of the systemic and local immunological responses of the feto-maternal unit that stimulate the pathway to sPTL and birth^10,11^. Therefore, identifying these unique cytokines and chemokines is crucial to understanding the pathogenesis of sPTB and biomarker discovery.

Since the inflammatory response triggered by dysregulated vaginal microbiome can continue undetected, it can possibly assume a chronic status mediated by the CXCR3 receptor chemokines (CXCL9, CXCL10 and CXCL11)^29,31^. This CXCR3-chemokine pathway is often implicated in chronic placental inflammation associated with maternal anti-foetal rejection and sPTB^29,31–37^, but are yet to be explored extensively in the CV space. We hypothesise that overexpression of CXCR3 ligands and other chemokines/cytokines in the CV space may be indicative of chronic inflammation in gestational tissues and metabolic fingerprints that may be linked with dysbiosis-, infection- and inflammation-induced sPTB.

This study explores how temporal changes in CV microbial and metabolite composition and function, and mediators of chronic inflammation are associated with sPTB. Therefore, using Nanopore Minion sequencing platform, we investigated changes in CV microbiota and metabolome across two gestational time points (GTPs) within the second trimester in asymptomatic women who experienced term or sPTB. We also analysed their associated metabolic pathways to gain insight into the likely pathomechanisms of sPTB. Furthermore, we measured temporal changes in the chemokines/cytokines CXCL9, CXCL10, CXCL11, TNF-α, eotaxin and TNFR1 to determine the role of the host immunological responses in inflammation-associated sPTB. This paper addresses mechanisms, pathways and insights into possible biomarkers for sPTB.

## Methods

### Study population

This is a prospective (longitudinal) cohort study of asymptomatic pregnant women at high risk of PTB based on a previous history of PTB or transvaginal ultrasound cervical length ≤ 25 mm (*n* = 49) recruited from the Jessop Maternity Wing of the Royal Hallamshire Hospital, Sheffield, UK (a tertiary PTB referral centre with circa 7500 births annually) between May 2018 and May 2019 after written informed consent was obtained. Pregnant women below 16 years of age or with uncertain gestation duration or multiple gestation, who presented with symptoms suggestive of preterm labour, preterm prelabour rupture of membranes (PPROM), or had a recent vaginal examination, evidence of genital tract infection (e.g. bacterial vaginosis, BV), urinary tract infection, vaginal bleeding, foetal anomaly, or abnormal cervical cytology were excluded from the study. Women with BV were excluded after BV screening because such infections can alter the vaginal microbiome and metabolite signature^38^. Women with no known risk of PTB, or taking antibiotics medication were also excluded from the study. The primary study outcome was spontaneous delivery before 37 completed weeks of gestation (PTB). As they were asymptomatic, the majority (~92%) of the participants did not receive any intervention, while three received progesterone and only one woman received a combination of progesterone and cerclage.

The study received approval by the Health Research Authority (HRA) and Health and Care Research Wales (HCRW) (REC reference18:/LO/2044).

### Sample collection

**Fig. 1:**
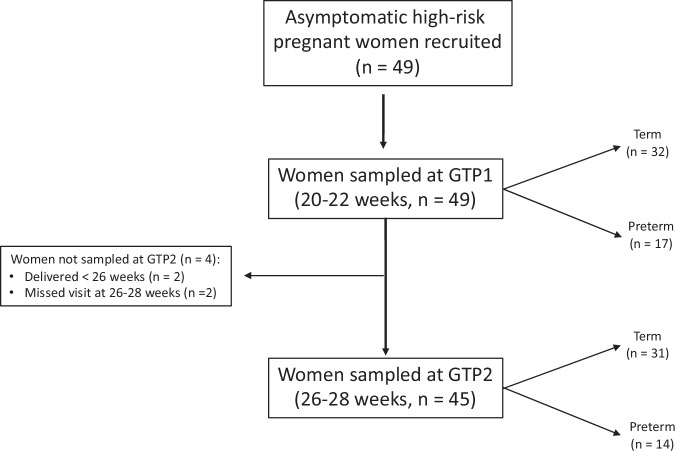
Study population and sample flow chart. GTP gestational time point.

CV fluid was collected from eligible study participants at two gestational time points (GTPs): GTP1 (20^+0^–22^+6^ weeks) *n* = 49; and a subset of this population was sampled again at GTP2 (26^+0^–28^+6^ weeks) *n* = 45 using the same previously described protocol^7–11^. Four women were not sampled again at GTP2 because two of them delivered before 26 weeks, while the other two missed the second antenatal visit at 26–28 weeks (Fig. 1). CV fluid samples were obtained before any vaginal examination or clinical intervention. The swabs were stored at −20 °C immediately after collection and later transferred to −80 °C within 1–3 days. After the collection of CV fluid samples, CV foetal fibronectin (FFN) concentration was also measured as previously reported^7–11,39^ to predict the risk of PTB^26^. Levels of CV fluid FFN > 50 ng/mL at or after 22 weeks gestation are associated with an increased risk of sPTB^26,27^.

### DNA extraction from swabs with CV fluid

All tips, tubes, tweezers and scissors were UV sterilised for 15 min prior to DNA extraction to reduce bacterial DNA contamination. To elute CV fluid for DNA extraction, the swab was thawed and 500 µL PBS was added and then vortexed for 5 min. The swab was placed into a new tube and centrifuged at 10,000 × *g* for 1 min to draw out any remaining fluid from the swab. The 500 µL of CV fluid was transferred to a clean tube and incubated with 75 µL of 20 mg/mL lysozyme (Fisher Scientific) at 37 °C for 1 h in order to degrade bacterial cell walls. DNA was extracted using the QIAmp DNA mini kit (Qiagen, UK, 51304) according to the manufacturer’s instructions adapting the protocol in our previous work^7^. Purified DNA was eluted from the QIAamp kit spin column in 50 μL of buffer AE and stored at −20 °C until further processing. The DNA sample was placed on ice prior to PCR amplification. In addition to the CV fluid samples, a blank swab also underwent DNA extraction to control for bacterial contaminants in reagents.

### 16S rRNA PCR and sequencing

PCR was performed with 20 µL of the extracted DNA on two separate occasions for each sample to ensure maximum output of amplified DNA and to reduce the chance of contamination. PCR conditions: 25 µL of Taq PCR master mix kit (Qiagen, UK) with final concentrations: MgCl_2_ 1.5 mM, 2.5 units Taq DNA polymerase, 200 µM of each dNTP; 5 µL of novel barcoded primers (0.2 µM each—*Forward* (5’–3’) 319F—CTCCTACGGGAGGCAGCAGT and *Reverse* (5’–3’) MCRevA—CTCACGACACGAGCTGACGAC); and 5 pg of bacterial DNA in TE buffer (as recommended by Qiagen). Details of the unique barcode sequences are in supplementary Table 1. A no-template control (NTC) was included for each pair in every PCR reaction. A DNA standard (Vaginal Microbiome Genomic Mix ATCC, MSA-1007) containing 16.7% *Gardnerella vaginalis* (ATCC 14019), 16.7% *Lactobacillus gasseri* (ATCC 33323), 16.7% *Mycoplasma hominis* (ATCC 23114), 16.7% *Prevotella bivia* (ATCC 29303), 16.7% *Streptococcus agalactiae* (ATCC BAA-611), 16.7% *Lactobacillus jensenii* (ATCC 25258) was used as a positive PCR control. The reaction conditions included an initial denaturation step of 94 °C for 3 min followed by 35 cycles of 94 °C for 45 s (denaturation), 60 °C for 1 min (annealing), 72 °C for 1 min 30 s (elongation), and a final extension of 72 °C for 10 min. The presence and size of the PCR amplicons were checked using 1.2% agarose gel electrophoresis containing GelRed nucleic acid stain (1:10,000) ran at 80 V for 1 h 30 min. The amplicons were purified using AMPure XP clean-up beads (Beckman, A63880). A ratio of 1.2 was used to remove DNA < 150 bp long. DNA was eluted in 12 µL of TE buffer. Amplicon (DNA) concentration was measured using Qubit dsDNA HS Assay Kit (Invitrogen, 10606433) and diluted to either 1.5 or 52 ng/sample, pooled and sequenced using the Oxford Nanopore MinION sequencing platform at the Sheffield Institute for Translational Neuroscience (SITraN), University of Sheffield, UK.

### Sequence analysis (microbiota profiling)

The raw FASTQ sequence files generated using Nanopore MinION sequencing technology were analysed using an in-house generated 16S data analysis pipeline. The analysis included demultiplexing and adaptor trimming using porechop v0.2.3, while quality check and filtering of low-quality and short reads were done using pycoQC v2.5.2. The median PHRED quality of reads score was 10.33 (>90% accuracy)^40,41^. Any score <8 was not considered for subsequent analysis. The median read length was 795.5 bp. The bacterial sequence reads were mapped against the SILVA database using QIIME2. The SILVA database was employed because it showed the highest number of bacterial species compared to Greengenes, RDP, NCBI and Ezbiocloud in our preliminary study (*unpublished data*). Hence, we concluded that it was more suitable in characterising the microbiota of this cohort as sequenced by Nanopore MinION.

We classified the vaginal communities into community state types (CSTs) as in our previous study^7^. That is, if at least half of the vaginal bacterial community is dominated by a particular species, then that species is regarded as dominant in that community. Furthermore, alpha diversity, which measures both the number of species and the inequality between species abundance was calculated using Shannon’s diversity index. The in-house pipeline calculates alpha diversity using an OTU table.

To determine significant differences in bacterial species composition, a comparison of bacterial abundance (CSTs) were made. Between the groups for whole samples, we compared preterm vs. term, and visit 1 vs. visit 2. At GTP1 (visit 1) and GTP 2 (visit 2), we compared preterm vs. term employing Wilcoxon Rank Sum test in *R* statistical tool (version 4.0.3). The data was analysed at specific GTPs because microbiota-metabolite and inflammatory responses may vary with gestation^7,11^.

### Metabolomic analysis

#### Sample preparation

The swabs were processed by suspension in 1 mL of isotonic phosphate-buffered saline (PBS). The 1.5 μL microfuge tube containing the cut end of the swab suspended in 1 mL PBS was vortexed for 5 min to elute the adsorbed CV fluid into the solution. The swab tip was safely discarded, and the remaining solution was centrifuged at 13,000 rpm for 3 min, after which the supernatant was aspirated into a clean tube and preserved at −80 °C until further analysis by liquid chromatography-mass spectrometry (LC–MS) for metabolomics and multiplexed bead-based immunoassay for cytokines. This protocol is similar to that employed in our previous publications^7–10,39,42^. As shown in our previous studies^7–10,39,42^, storage of CV fluid samples in either −20 °C or later in −80 °C or processing immediately after collection from patients does not affect metabolite concentrations^43^.

All samples (100 μL) were centrifuged at 12,000 rpm for 2 min and then diluted 1:10 with 50:50 methanol:water. The methanol was LC–MS grade (Honeywell) and the water was ultrapure water from an ELGA water purification system. The samples were analysed to obtain a metabolite fingerprint in both positive (detects peaks corresponding to protonated analytes) and negative (detects peaks corresponding to deprotonated analytes) ionisation modes by directly injecting 10 μL into a Waters GSi Synapt time of flight (TOF) mass spectrometer (Waters, UK) using electrospray (ESI) ionisation. Positive mode conditions used were capillary 2.6 kV, sample cone 80 V, source temperature 100 °C, desolvation temperature 280 °C and desolvation gas flow 500 L/h. Negative conditions used were capillary 2 kV, sample cone 20 V, source temperature 100 °C, desolvation temperature 280 °C and desolvation gas flow 650 L/h. The injection was automated using a Waters Acquity UPLC system. Data was collected in continuum mode over the mass range 50–800*m*/*z* with a scan time of 1 s per scan. Each sample was run in triplicate and these replicates were converted to lists of *m*/*z* vs. ion intensity and combined using an in-house Excel macro based on ref. ^44^ for noise reduction and binning of data to 0.2 amu-sized bins. The data was normalised to the total ion count. The macro also assigned putative IDs by matching accurate mass to the Humancyc database—the encyclopaedia of genes and metabolism (https://humancyc.org/) using a tolerance of 20 ppm. IDs were cross-checked against the HMDB human metabolome database (https://hmdb.ca/).

Multivariate principal component analysis (PCA) and orthogonal partial least squares discriminant analysis (OPLS-DA) analyses were performed using SIMCA 15.0.2, and pathway analysis was performed using Metaboanalyst 5.0 using the MetaboanalystR package (https://www.metaboanalyst.ca/)^45^. Compound lists using putatively identified metabolites were used as annotated features and submitted online to the Metaboanalyst pathway analysis option.

### CV fluid chemokine/cytokine profiling

The concentrations of six CV fluid pro-inflammatory chemokines/cytokines, including CXCL9, CXCL10, CXCL11, TNF-α, eotaxin and TNFR1 were determined using a multiplexed bead-based immunoassay (BD^TM^ Cytometric Bead Array (BD Biosciences, CA, USA)). The analysis was performed using 50 μL of CV fluid sample and according to the instructions of the BD cytometric bead array (CBA) Human Enhanced Sensitivity Master Buffer Kit (Cat. No. 561521) and as formerly described^10,11,46^. Briefly, the assay involved the use of cytokine-specific capture beads and detector antibodies (Detection reagent Parts A and B) with fluorescent characteristics. These capture beads and detection antibodies form sandwich or organometallic complexes with the standards (Human Flex Set Standards) and cytokines in the unknown samples when incubated together at room temperature away from ultraviolet light. The emergent fluorescence from these complexes is proportional to the concentration of bound cytokines and is measured by flow cytometry. The mean fluorescent intensities (MFI) of both standards and unknowns were presented graphically using the FCAP Array^TM^ software (version 1.0). The final concentrations (pg/ml) of the cytokines were extrapolated from the calibration curve produced on GraphPad Prism 9.2.0 (*RIA or ELISA—Interpolate unknowns from sigmoidal curve*) using the concentrations and MFIs of the standards. The samples were analysed at random by an operator unaware of the delivery outcomes of the study participants. The BD CBA Human Enhanced Sensitivity Flex Set assays are more sensitive than most corresponding ELISAs and permit multiplexed analysis of several cytokines from a single sample in less time. A detailed description of this procedure can be found in Supplementary Note 1.

### Statistical analyses

The identified metabolite data matrices were analysed according to GTPs and delivery outcome (preterm vs. term) using the Omu metabolomics analysis *R* package in *R* version (4.0.3). The Omu package was used to assign metabolic pathways (carbohydrates, lipids, vitamins, etc.) which are included as metadata. The analysis involves performing Welch’s *t*-tests, ANOVAs and principal component analysis (PCA), and gathering functional orthology and gene names from the Kyoto Encyclopaedia of Genes and Genomes (KEGG) metabolic pathway database that are associated with the metabolites in a dataset. The univariate statistical models, *t*-test and ANOVA were generated using the functions omu_summary and anova_function, respectively. Both functions provide outputs of *p* values and adjusted *p* values, while omu_summary provides output of group means, standard error, standard deviation, fold change, and log2foldchange. Both of these models were useful for observing relationships between independent variables in this experiment/dataset. The data frame created using the assign_hierarchy function was used in the count_data argument of omu_summary to run statistics on it. The output of omu_summary was used for visualisation plots. To identify differences in metabolite normalised % total ion count between (preterm vs. term) and within (GTP1 vs. GTP2) the groups, a false discovery rate (FDR) correction was performed using Benjamini–Hochberg and selected a 5% FDR cut-off (0.05) and fold change cut off ±1. Gathering functional orthology and gene data was also performed. The analysis retrieved data from the KEGG API using the KEGGREST *R* package. This analysis identified genes associated with the functional orthologs for all organisms (human, bacteria). It was useful to identify genes and hierarchical class assignments for using metabolomics to screen for a hypothesis, in order to study organisms via a reductionist approach. It was also useful to find compounds that changed between experimental groups, and then identify genes in an organism of interest involved in enzymatic reactions with the compounds that changed significantly.

To determine the changes of inflammatory biomarkers across the mid-trimester, the concentrations of pro-inflammatory cytokines were compared between term and preterm-delivered women by Wilcoxon rank-sum test in each GTP and as ratios of GTP1/GTP2 for women who provided samples at both GTPs. The distribution of the data was determined by the Shapiro–Wilk normality test. *P* values < 0.05 were considered statistically significant. Predictive capacities of the chemokines/cytokines for sPTB were determined by binary logistic regression models and receiver operating characteristics (ROC) curves to determine the specificity, sensitivity and area under the ROC curve (AUC). Multiple regression analysis was performed to determine whether the combination of two or more analytes or clinically-used biomarkers such as FFN can improve the prediction of sPTB. The chemokines/cytokines were also correlated with FFN using Spearman’s correlation coefficient (*ρ*). All analyses were performed using MedCalc Version 20 (MedCalc Software bvba, BE) and GraphPad Prism 9.2.0 (GraphPad Software, Inc., CA, USA) statistical software packages.

### Integrative profiling of microbiota, metabolome and cytokines

Metabolome, microbiota and cytokines data were analysed together using the *R* statistical tool (version 4.0.3) in a subset of women/samples. The significant metabolites, microbiota (CSTs and relative abundance) and cytokine data were tested using Pearson’s correlation coefficient (*r*) to observe any significant positive and negative correlations within and between the groups i.e. GTP1 + GTP2, GTP1: preterm+term, and GTP2: preterm+term.

## Results

Overall, the study participants were predominantly whites (83.7%), while others were Asians (12.2%), Blacks (2.0%) and Hispanics (2.0%). These percentages were reduced after the overall study population was subdivided into GTPs and preterm vs. term groups (Table 1). The median cervical length was 36 ± 5.6 mm, and only four participants had short cervix (≤25 mm). The PTB rate was 34.7% (17/49), and 23.5% of the women who delivered preterm experienced preterm prelabour rupture of membranes (PPROM) before delivery. Maternal age, body mass index (BMI), and CV FFN concentration did not differ significantly between the term- vs. preterm-delivered women. The participant’s demographic and clinical details are presented in Table 1.

**Table 1:** Demographic and clinical details

Characteristics	GTP1 (20–22 weeks)		GTP2 (26–28 weeks)	
	Term (*n* = 32)	Preterm (*n* = 17)	Term (*n* = 31)	Preterm (*n* = 14)
Age, years	31.28 ± 5.83	28.76 ± 6.09	31.65 ± 6.06	29.71 ± 5.54
BMI, kg/m^2^	26.80 ± 5.84	26.18 ± 4.68	27.27 ± 6.28	26.94 ± 4.05
Smoker, *n* (%)	8(25)	1(5.9)	7(22.6)	1(7.1)
FFN, ng/mL	4.0 ± 1.80	4.35 ± 0.93	3.65 ± 1.40	3.29 ± 1.64
GAP, weeks	20.9 ± 1.12	20.0 ± 0.94**	26.7 ± 0.96	26.1 ± 1.30
GAD, weeks	39.0 ± 1.17	32.6 ± 4.04****	39.0 ± 1.21	33.4 ± 3.07****
PPROM, *n* (%)	NA	4 (23.5)	NA	3 (21.4)
*Race/ethnicity, n (%)*				
White	27 (84.4)	14 (82.4)	26 (83.9)	12 (85.7)
African	1 (3.1)	–	1 (3.2)	–
Asian	3 (9.4)	3 (17.6)	3 (9.7)	2 (14.3)
Hispanic	1 (3.1)	–	1 (3.2)	–

Data is presented as mean ± standard deviation. The women in GTP2 are a subset of those in GTP1, hence the similarity in demographic/clinical details. The reduced sample population at GTP2 is because two women delivered before 26 weeks (GTP2), while another two women missed the second study visit, hence, their samples were not obtained. Proportion of whites = 83.7% in the total study population.

*FFN* foetal fibronectin, *GAD* gestational age at delivery, *GAP* gestational age at presentation, *GTP* gestational time point, *PPROM* preterm prelabour rupture of membranes, *NA* not applicable.

**P < 0.01, ****P < 0.0001.

### Microbiota profiling

To characterise the CV microbiota composition according to delivery outcomes, determine any changes in the microbial community with gestation, and determine the probable source of the observed metabolite changes, we sequenced the full length of the bacterial 16S rRNA genes in a subset of 40 CV fluid samples (GTP1 = 21 and GTP2 = 19; term = 26 and preterm = 14). Sequencing the full length of the bacterial DNA provides wider data (sequence) coverage that includes all regions, and is an improvement from our previous study^7^ and others^1,6,20,47^ that sequenced only predetermined variable regions of interest.

### Assignment of community state types

Using our previous classification method^7^, we observed that among all the samples combined, *Lactobacillus*_unclassified (unidentified *Lactobacillus* species, 32.5%) was the most abundant, followed by CSTIVB (mixed anaerobes with unidentified species, 17.5%), CSTIVA (mixed anaerobes, i.e. *Gardnerella vaginalis*, *Fannyhessea vaginae*, *Bifidobacterium* spp., *Gemella asaccharolytica*, *Mobiluncus curtisii*, *Mycoplasma hominis*, *Prevotella amnii*, *Prevotella bivia*, *Ureaplasma urealyticum*, *Ureaplasma parvum*, *Sneathia amnii, Streptococcus agalactiae*, *S. pneumoniae, Parvimonas, Campylobacter, Peptoniphilus, Pseudomonas,* etc. 15%), CST* (*Lactobacillus vaginalis, Lactobacillus delbrueckii*, *Lactobacillus helveticus, Lactobacillus rhamnosus, Lactobacillus acidophilus,* etc. 10%), CSTIII (*Lactobacillus iners*, 10%), CSTI (*Lactobacillus crispatus*, 2.5%), and CSTV (*Lactobacillus jensenii*, 2.5%). Furthermore, some samples showed two dominant CSTs at an equal amount, defined as *Lactobacillus*_unclassified/CST* (2.5%), *Lactobacillus*_unclassified/CSTIVB (2.5%), CSTIII/*Lactobacillus*_unclassified (2.5%) and CST IVA/CSTIVB (2.5%). Significant amount of CSTII dominated by *L. gasseri* was not identified in this population. Furthermore, we combined GTP1 and GTP2 (Table 2) to determine the prevalent bacterial species during pregnancy irrespective of gestational age at sampling. *F. vaginae, G. vaginalis, R. rhizogenes*, *C. bacterium* DNF00809, *S. pneumonia*, and *C. trachomatis* were more prevalent in the women who delivered preterm than their term-delivered counterparts (*χ*^2^ test = 26.01, *p* < 0.0001, Table 2).

**Table 2:** Most prevalent bacterial species identified from cervicovaginal fluid samples of asymptomatic pregnant women at risk of preterm birth at both gestational time-points

Taxon	Prevalence in samples (%)	
	Term (*n* = 26)	Preterm (*n* = 14)
*Fannyhessea vaginae*	11.5	35.7
*Gardnerella vaginalis* 1400E	15.4	57.1
*Coriobacteriales bacterium* DNF00809	0	28.6
*R. rhizogenes*	23.1	64.3
*Streptococcus pneumoniae*	38.5	50.0
*Lactococcus lactis subsp. cremoris*	42.3	42.9
*Lactobacillus jensenii*	38.5	42.9
*Chlamydia trachomatis*	26.9	42.9
*Lactobacillus iners*	46.2	42.9
*Lactobacillus crispatus*	26.9	50.0
*Lactobacillus helveticus*	15.4	42.9
*Lactobacillus acidophilus*	19.2	35.7
*Lactobacillus plantarum*	11.5	35.7
*Lactobacillus* sp. KC38	30.8	35.7
*Idiomarina* sp. P7-5-3	26.9	21.4

A total of 40 cervicovaginal fluid samples (GTP1: 20–22 weeks = 21 and GTP2: 26–28 weeks = 19). Significance of the prevalence of bacterial species in preterm vs. term women was determined by chi-squared test.

*GTP* gestational time point.

### Metabolite profiling

A total of 1842 metabolites were identified from 94 CV fluid samples obtained at GTP1 (*n* = 49) and GTP2 (*n* = 45).

### Microbiota-metabolite and chemokine/cytokine profiles at GTP1

**Fig. 2:**
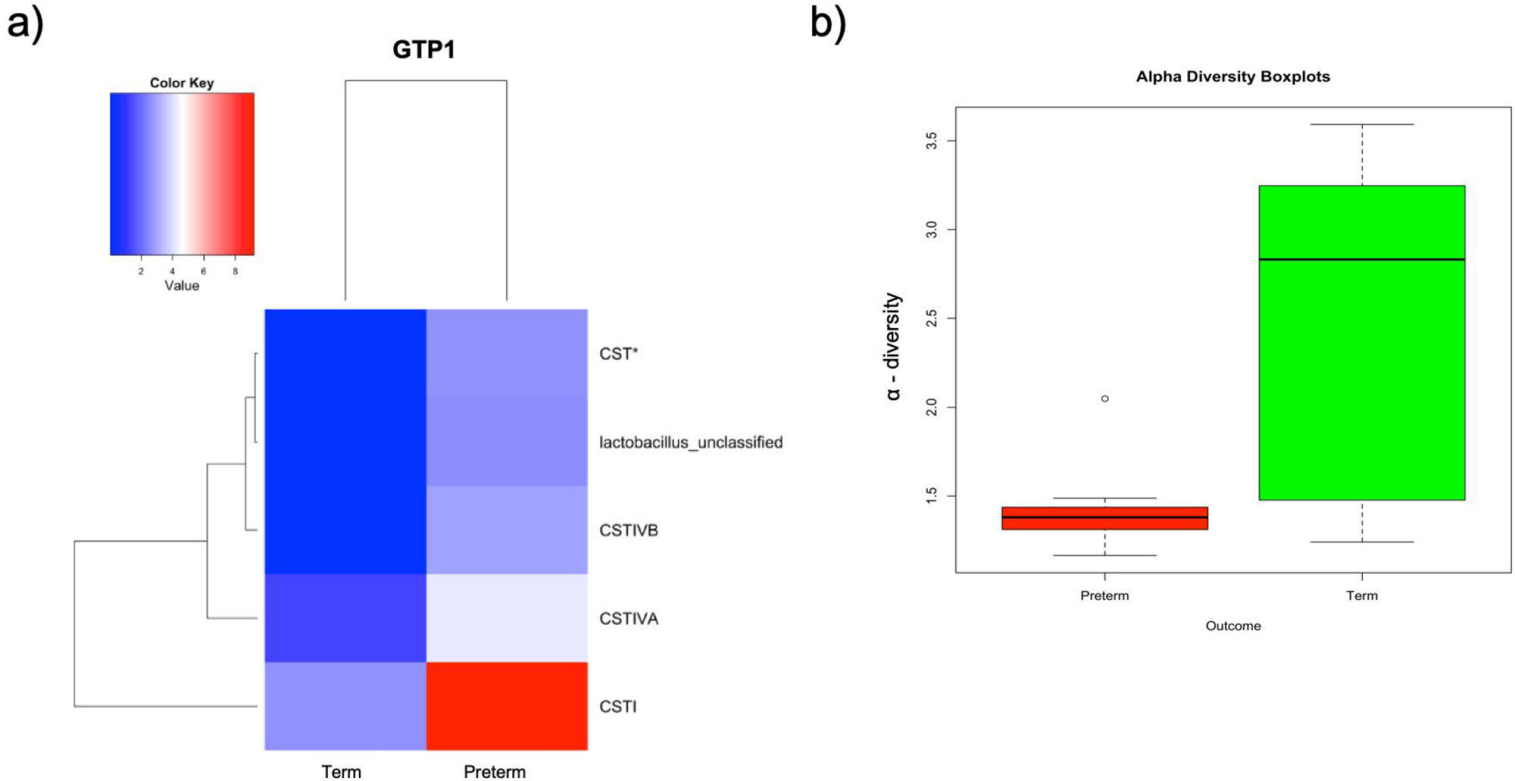
Changes in cervicovaginal bacterial community at gestational time point (GTP) 1 (20–22 weeks). **a** Community state types (CSTs) and **b** Alpha-diversity. *Key*: red = high or more dominant; blue = low or less dominant. Term = 13 vs. preterm = 8. Wilcoxon rank-sum test, *p* < 0.0001.

**Fig. 3:**
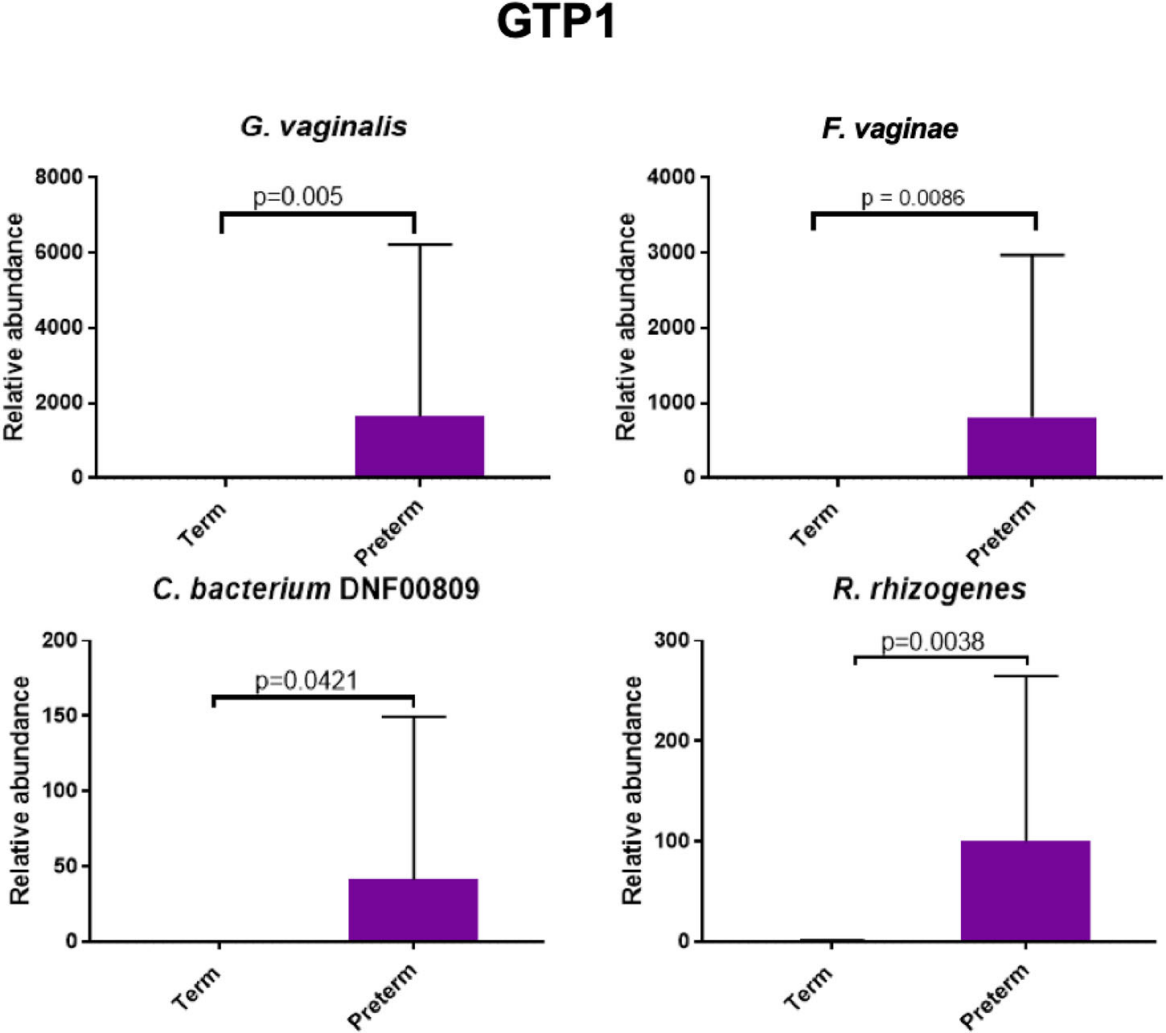
Comparison of bacterial species relative abundance at the gestational time point (GTP) 1 (20–22 weeks). The bacterial species relative abundances in the term group were very low (zero in most cases) relative to the preterm group, hence, the bars are almost absent. Term = 13 vs. preterm = 8. Wilcoxon rank-sum test, bars represent mean, error bars represent 95% confidence interval.

**Fig. 4:**
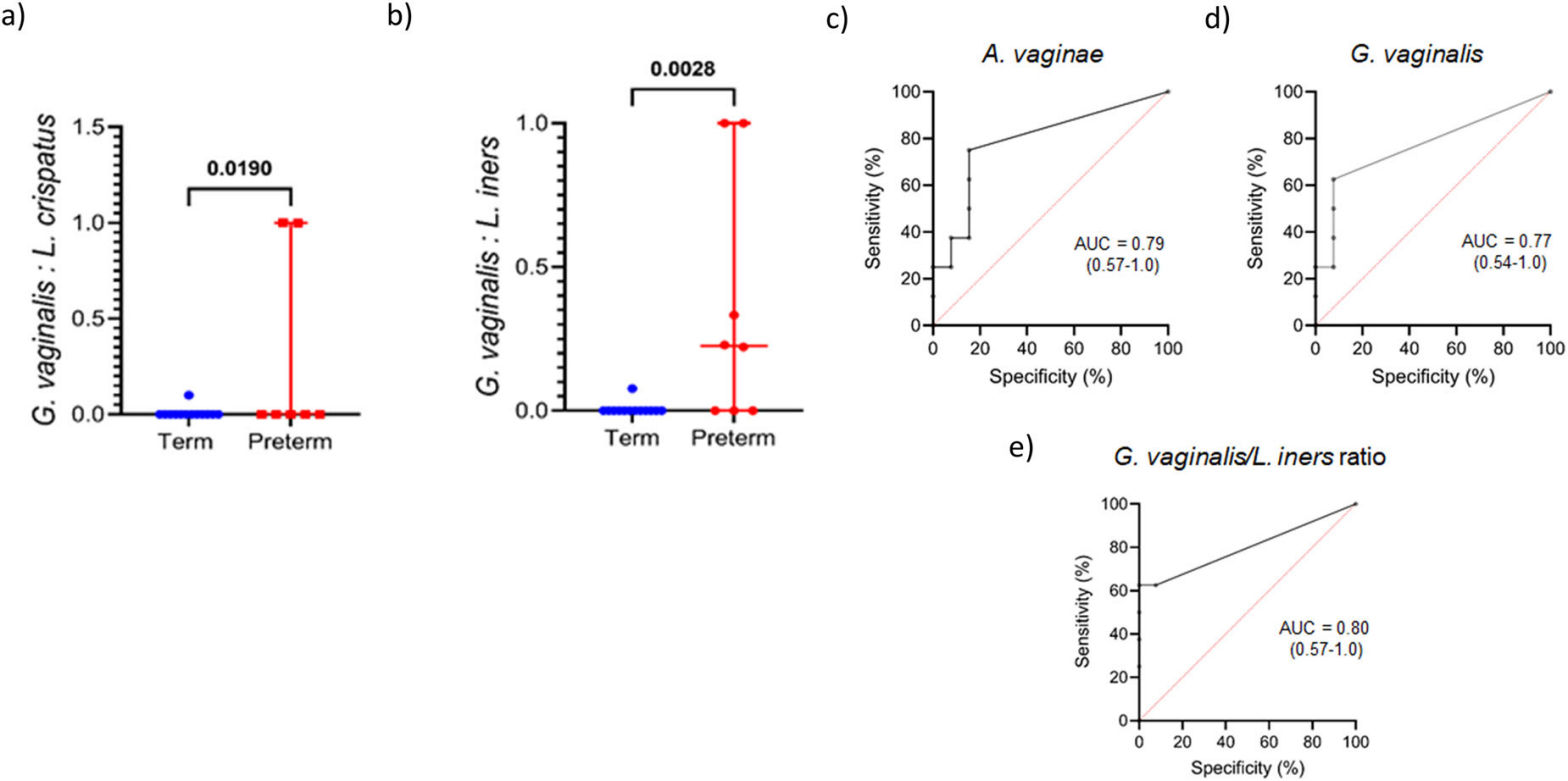
Relative abundance of clinically relevant cervicovaginal bacterial species at GTP1 (20-22 weeks). **a** *Gardnerella vaginalis/Lactobacillus crispatus* ratio. **b** *Gardnerella vaginalis/Lactobacillus iners* ratio (middle line = median, error bars = 95% Confidence Interval, Wilcoxon rank-sum test). **c–e** Receiver operating characteristic curve analysis of cervicovaginal bacterial species relative abundance for the prediction of spontaneous preterm birth. Term = 13; Preterm = 8. AUC area under receiver operating characteristic curve, GTP gestational time point.

At GTP1, the preterm-delivered women showed more CSTI, CSTIVA, CSTIVB, CST* and *Lactobacillus*_unclassified compared to the term-delivered women (Fig. 2a). The bacterial community diversity depicted by alpha-diversity was higher in the term- than preterm-delivered women (*p* < 0.0001, Fig. 2b). Only *G. vaginalis* (*p* = 0.005)*, F. vaginae* (*p* = 0.0086)*, Coriobacteriales bacterium* DNF00809 (*p* = 0.0421), and *R. rhizogenes* (*p* = 0.0038) were more abundant in the women who delivered preterm compared to their term-delivered counterparts at GTP1 (term *n* = 13 vs. preterm *n* = 8) (Fig. 3). There were 17 and 30 significant positive correlations between the most abundant bacterial species in the term and preterm groups respectively. No negative correlation between the species was observed in any of the groups (Table 3). Because *L. crispatus* abundance is believed to be associated with low risk of PTB, while *L. iners* and *G. vaginalis* which often coexist at high frequencies are association with increased risk of PTB^48^, we compared the ratios of *G. vaginalis/L. crispatus, G. vaginalis/L. iners*, and *L. iners/L. crispatus* in relation to birth outcome. There was a significantly higher relative abundance of *G. vaginalis* compared to *L. crispatus* (p = 0.019) and *L. iners* (p = 0.0028) in the preterm group at GTP1 only (Fig. 4a, b). Furthermore, ROC curves of bacterial species relative abundance revealed that *F. vaginae* (AUC = 0.79), *G. vaginalis* (AUC = 0.77) and *G. vaginalis/L. iners* ratio (AUC = 0.80) significantly identified women at risk of sPTB at GTP1 only with high sensitivities, specificities and likelihood ratios (Table 4 and Fig. 4c–e).

**Table 3:** Correlation of cervicovaginal bacterial species at different gestations according to delivery outcome

	Pearson’s correlation (*r*)	*p*-value
GTP 1 (20–22 weeks)		
Term (*n* = 13)		
* Fannyhessea vaginae* vs. *Gardnerella vaginalis*	0.677	0.011
* Fannyhessea vaginae* vs. *Megasphaera* unidentified	1.000	0.000
* Chlamydia trachomatis* vs. *Lactobacillus acidophilus*	0.887	<0.000
* Chlamydia trachomatis* vs. *Lactobacillus crispatus*	0.999	0.000
* Chlamydia trachomatis* vs. *Lactococcus lactis*	0.807	0.001
* Gardnerella vaginalis* vs. *Megasphaera* unidentified	0.677	0.011
* Gardnerella vaginalis* vs. *Shuttleworthia* unidentified	0.793	0.001
* Lactobacillus jensenii* vs. *Lactobacillus iners*	0.853	<0.000
* Lactobacillus jensenii* vs. *Rhizobium rhizogenes*	0.739	0.004
* Lactobacillus jensenii* vs. *Streptococcus pneumoniae*	0.888	<0.000
* Lactobacillus crispatus* vs. *Lactococcus lactis*	0.813	0.001
* Lactobacillus crispatus* vs. *Lactobacillus acidophilus*	0.901	<0.000
* Lactococcus lactis* vs. *Streptococcus pneumoniae*	0.798	0.001
* Lactococcus lactis* vs. *Lactobacillus acidophilus*	0.843	<0.000
* Lactobacillus iners* vs. *Rhizobium rhizogenes*	0.609	0.027
* Lactobacillus iners* vs. *Streptococcus pneumoniae*	0.850	<0.000
* Rhizobium rhizogenes* vs. *Streptococcus pneumoniae*	0.621	0.024
Preterm (*n* = 8)		
*F. vaginae* vs. *Coriobacteriales bacterium*	1.000	<0.000
*F. vaginae* vs. *G. vaginalis*	1.000	<0.000
*F. vaginae* vs. *Megasphaera* unidentified	0.894	0.003
*F. vaginae* vs. *Prevotella amnii*	0.999	<0.000
*F. vaginae* vs. *R. rhizogenes*	0.920	0.001
*C. trachomatis* vs. *L. crispatus*	1.000	0.000
*C. trachomatis* vs. *L. lactis*	1.000	<0.000
*C. trachomatis* vs. *S. pneumoniae*	0.962	<0.000
*C. trachomatis* vs. *Idiomarina* sp. P7-5-3	0.991	<0.000
*C. trachomatis* vs. *L. acidophilus*	0.940	0.001
*G. vaginalis* vs. *C. bacterium*	0.999	<0.000
*G. vaginalis* vs. *Megasphaera* unidentified	0.886	0.003
*G. vaginalis* vs. *P. amnii*	1.000	<0.000
*G. vaginalis* vs. *R. rhizogenes*	0.913	0.002
*L. crispatus* vs. *L. lactis*	1.000	<0.000
*L. crispatus* vs. *S. pneumoniae*	0.962	<0.000
*L. crispatus* vs. *Idiomarina* sp. P7-5-3	0.991	<0.000
*L. crispatus* vs. *L. acidophilus*	0.940	0.001
*L. lactis* vs. *S. pneumoniae*	0.963	<0.000
*L. lactis* vs. *Idiomarina* sp. P7-5-3	0.989	<0.000
*L. lactis* vs. *L. acidophilus*	0.938	0.001
*Idiomarina* sp. P7-5-3 vs. *L. acidophilus*	0.944	<0.000
*Megasphaera* unidentified vs. *C. bacterium*	0.905	0.002
*Megasphaera* unidentified vs. *P. amnii*	0.882	0.004
*Megasphaera* unidentified vs. *R. rhizogenes*	0.988	<0.000
*P. amnii* vs. *C. bacterium*	0.998	<0.000
*P. amnii* vs. *R. rhizogenes*	0.909	0.002
*R. rhizogenes* vs. *C. bacterium*	0.929	0.001
*S. pneumonia* vs. *Idiomarina* sp. P7-5-3	0.950	<0.000
*S. pneumonia* vs. *L. acidophilus*	0.991	<0.000
GTP2 (26–28 weeks)		
Term (*n* = 13)		
*C. trachomatis* vs. *L. jensenii*	0.961	<0.000
*C. trachomatis* vs. *L. crispatus*	0.964	<0.000
*C. trachomatis* vs. *Shuttleworthia* unidentified	0.812	0.001
*C. trachomatis* vs. *L. acidophilus*	0.742	0.004
*G. vaginalis* vs. *Megasphaera* unidentified	0.995	<0.000
*L. jensenii* vs. *L. crispatus*	0.991	<0.000
*L. jensenii* vs. *Shuttleworthia* unidentified	0.914	<0.000
*L. jensenii* vs. *L. acidophilus*	0.699	0.008
*L. crispatus* vs. *Shuttleworthia* unidentified	0.893	<0.000
*L. crispatus* vs. *L. acidophilus*	0.705	0.007
*L. lactis* vs. *R. rhizogenes*	0.856	<0.000
*L. lactis* vs. *S. pneumoniae*	0.857	<0.000
*L. iners* vs. *S. pneumoniae*	0.645	0.017
*Shuttleworthia* unidentified vs. *L. acidophilus*	0.605	0.029
Preterm (*n* = 6)		
*F. vaginae* vs. *C. bacterium*	0.997	<0.000
*F. vaginae* vs. *Megasphaera* unidentified	0.994	<0.000
*C. trachomatis* vs. *L. crispatus*	1.000	<0.000
*C. bacterium* vs. *G. vaginalis*	0.857	0.029
*C. bacterium* vs. *Megasphaera* unidentified	0.985	0.000
*F. vaginae* vs. *G. vaginalis*	0.894	0.016
*G. vaginalis* vs. *Megasphaera* unidentified	0.927	0.008
*L. jensenii* vs. *L. iners*	1.000	<0.000
*L. jensenii* vs. *Shuttleworthia* unidentified	0.996	<0.000
*L. jensenii* vs. *S. pneumoniae*	0.988	<0.000
*L. jensenii* vs. *L. acidophilus*	1.000	<0.000
*L. lactis* vs. *L. iners*	0.874	0.023
*L. lactis* vs. *Shuttleworthia* unidentified	0.898	0.015
*L. lactis* vs. *S. pneumoniae*	0.938	0.006
*L. lactis* vs. *L. acidophilus*	0.888	0.018
*L. iners* vs. *Shuttleworthia* unidentified	0.996	<0.000
*L. iners* vs. *S. pneumoniae*	0.988	<0.000

*GTP* gestational time point.

**Table 4:** Prediction of preterm birth by vaginal bacterial species relative abundance at 20-22 weeks (GTP1)

	AUC (95% CI)	Sensitivity (%) (95% CI)	Specificity (%) (95% CI)	Positive likelihood ratio
*F. vaginae*	0.79 (0.57–1.0)	75 (41–96%)	85 (58–97%)	4.9
*G. vaginalis*	0.77 (0.54–1.0)	63 (31–86%)	92 (67–100%)	8.1
*G. vaginalis/L. iners* ratio	0.80 (0.57–1.0)	63 (31–86%)	92 (67–100%)	8.1

Term = 13; Preterm = 8.

*AUC* area under receiver operating characteristic curve, *CI* confidence interval, *GTP* gestational time point.

**Fig. 5:**
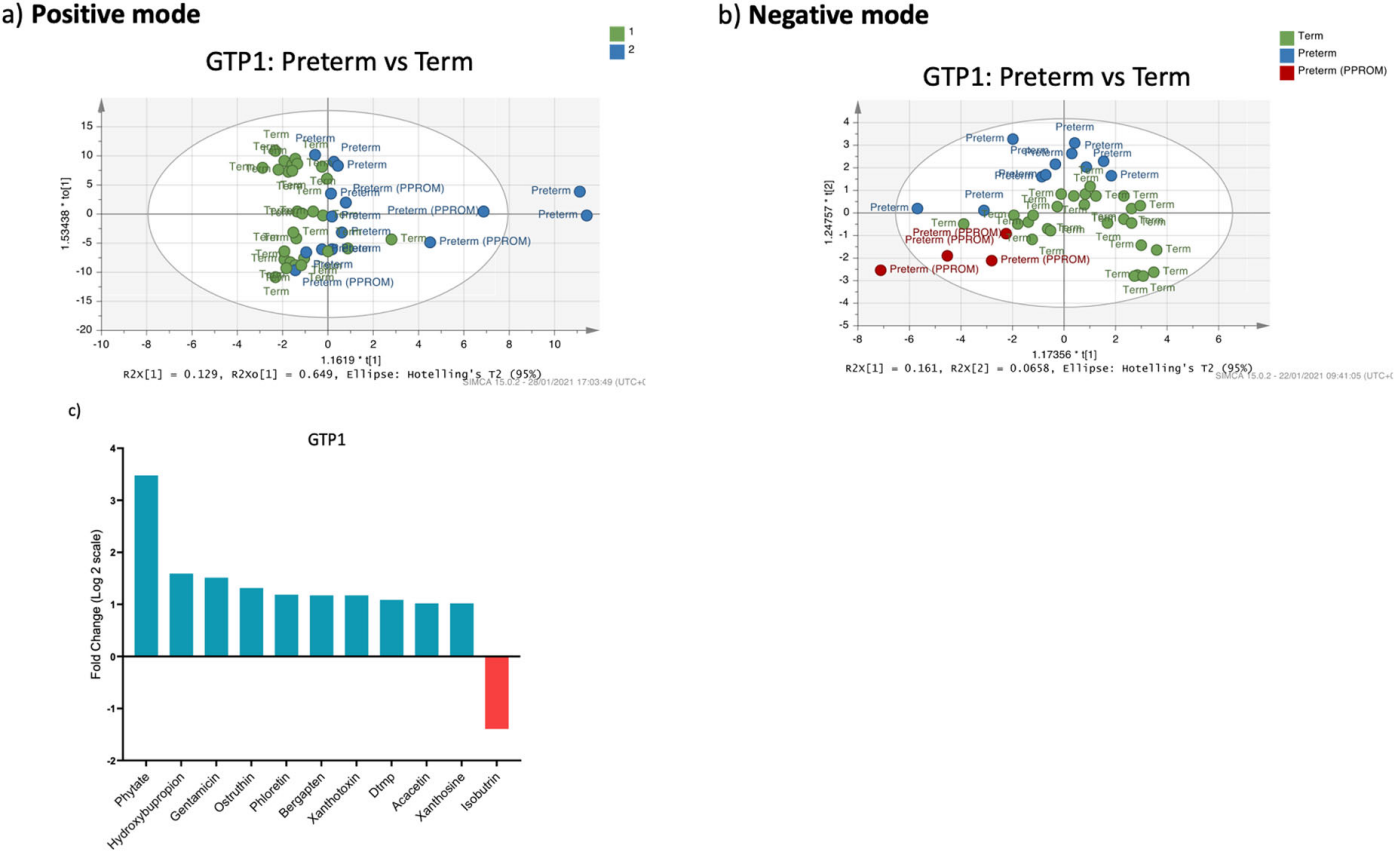
Orthogonal projections to latent structures discriminant analysis (OPLS-DA) of cervicovaginal fluid samples of preterm- and term-delivered women at gestational time point (GTP) 1 (20–22 weeks, *n* = 49). **a** Positive ionisation mode that detects peaks corresponding to protonated metabolites. **b** Negative ionisation mode, which detects peaks corresponding to deprotonated metabolites. **c** Fold change of metabolites that differ between preterm- vs. term-delivered women at GTP1. All the metabolites were upregulated in the women who delivered preterm except isobutrin (Welch’s *t*-test).

Furthermore, multivariate analysis of metabolite normalised % total ion count by OPLS-DA showed no clear separation of the samples between the term and preterm groups at GTP1 in the positive and negative ionisation modes that detect peaks corresponding to protonated and deprotonated analyte molecules, respectively (Fig. 5a, b). However, 20 metabolites differed in the term vs. preterm groups at GTP1. These were reduced to 11 metabolites when we applied a fold change (FC = Preterm−Term) cut-off of ±1. That is, only metabolite differences between the term and preterm groups that were >1 were considered significant and included in subsequent analysis and interpretation. Hence, at GTP1, only acacetin, bergapten, deoxythymidine monophosphate, gentamicin, hydroxybupropion, ostruthin, phloretin, phytate, xanthosine, xanthotoxin were significantly higher in the preterm- than term-delivered women, while isobutrin was lower (Table 5 and Fig. 5c).

**Table 5:** Fold change of cervicovaginal fluid metabolites that differ in preterm- vs. term-delivered women at GTP1 (20-22 weeks)

Metabolic pathway	Sub-pathway	Metabolite	Preterm/Term fold change (Log2)	*p*-value	KEGG ID	Function
Alcohols and polyols	Inositol phosphate metabolism; Phosphatidylinositol signalling system	Phytate	3.477	0.030	C01204	• Complexing agent for the removal of traces of heavy metal ions. • Hypocalcemic agent. • Strong chelator of important minerals such as calcium, magnesium, iron, and zinc • Contribute to mineral deficiencies in developing countries • Antioxidant and anti-inflammatory^68–70^
Carbonyl compounds	Alkyl-phenyl ketones	Hydroxybupropion	1.591	0.016	NA	Has pro-inflammatory effect at high doses^77^
Carbohydrates and carbohydrate conjugates	Aminocyclitol glycosides	Gentamicin	1.513	0.034	C00505	• Gentamicin action pathway • Directly involved in growth, development or reproduction • Anti-inflammatory action^75^
Phenylpropanoids	Hydroxycoumarins	Ostruthin	1.315	0.031	NA	• Inhibits LPS-induced iNOS and COX-2 expression • Reduces nitric oxide and PGE_2_ production. • Antimycobacterial activity • Used to treat inflammatory diseases. • Attenuates the up-regulation of ICAM-1 mRNA expression and inhibits NF-κB^73,74^.
	Furanocoumarins	Bergapten	1.173	0.035	C01557	• Decreases NF-κB, COX-2, iNOS^72^
		Xanthotoxin	1.173	0.035	C01864	• Reduces iNOS and COX-2 and production of NO and PGE_2_. • Reduces TNF-α and IL-6 production and expression • Inhibits the activation of AP-1 and NF-κB subunits. • Increases IkB^71^.
Lipids	Flavonoids	Phloretin	1.185	0.018	C00774	• Anti-inflammatory agent similar to NSAIDs • Employed in the treatment of vaginal inflammation (vaginitis)^66^ • Inhibit cell growth and inducing apoptosis • Inhibitor of eukaryotic urea transporters, blocks VacA-mediated urea and ion transport^116–118^ • a metabolite of *Escherichia*^119^
		Acacetin	1.020	0.031	C01470	• Inhibits macrophage infiltration and production of NO, iNOS and COX-2 in LPS-induced macrophage cells. • Tocolytic agent to prevent premature labour^64,65^
		Isobutrin	−1.393	0.020	C08649	• Hepatoprotective and anti-inflammatory agent • Inhibit PGE_2_ and MMP 1, 2, 9 and 10^81,82,83^
Nucleotides	Pyrimidine metabolism	DTMP	1.088	0.017	C00364	• DNA formation^79,80^
Alkaloids	Purine alkaloid biosynthesis; Purine metabolism	Xanthosine	1.020	0.031	C01762	• RNA damage product probably found at sites of inflammation by nucleobase deamination^76^

*p*-values are false discovery rate (FDR) adjusted.

*COX-2* cyclooxygenase-2, *DNA* deoxyribonucleic acid, *DTMP* deoxythymidine monophosphate, *GTP* gestational time point, *ICAM-1* intercellular adhesion molecule 1, *IkB* inhibitor of kappa-light-chain-enhancer of activated B cells, iNOS inducible nitric oxide synthase, *KEGG* Kyoto Encyclopaedia of Genes and Genomes, *LPS* lipopolysaccharide, *MMP* matrix metalloproteinase, *NF-κB* nuclear factor kappa-light-chain-enhancer of activated B cells, *NO* nitric oxide, *NSAIDs* nonsteroidal anti-inflammatory drugs, *PGE2* prostaglandin E2, *RNA* ribonucleic acid.

In order to determine whether the microbiota-metabolite changes are associated with a chronic subclinical inflammatory state, the concentrations of pro-inflammatory cytokines (CXCL9, CXCL10, CXCL11, TNF-α, eotaxin, TNFR1) in mid to late second trimester between term- and preterm-delivered women were determined. Out of the six chemokines/cytokines, CXCL11, TNF-α and eotaxin (in all samples) and TNFR1 (70% of samples) were below the detection limit of the assay, hence, their concentrations could not be extrapolated from the standard curve and were not included in subsequent analysis. On the other hand, CXCL9, CXCL10 (in all samples) and TNFR1 (30% of samples) were detected. However, none of these detected proteins differed between the term vs. preterm groups at GTP1. There were also no significant correlations between the microbiota, metabolites and chemokines/cytokines in the term-delivered women. Similar analysis could not be conducted in the preterm-delivered women due to the small sample numbers in this group. However, when the women were combined (irrespective of delivery outcomes), there were significant correlations between CSTs, bacterial species, metabolites, and chemokines/cytokines (Supplementary Note 2 and Figs. 1, 2).

### Microbiota-metabolite and chemokine/cytokine profiles at GTP2

**Fig. 6:**
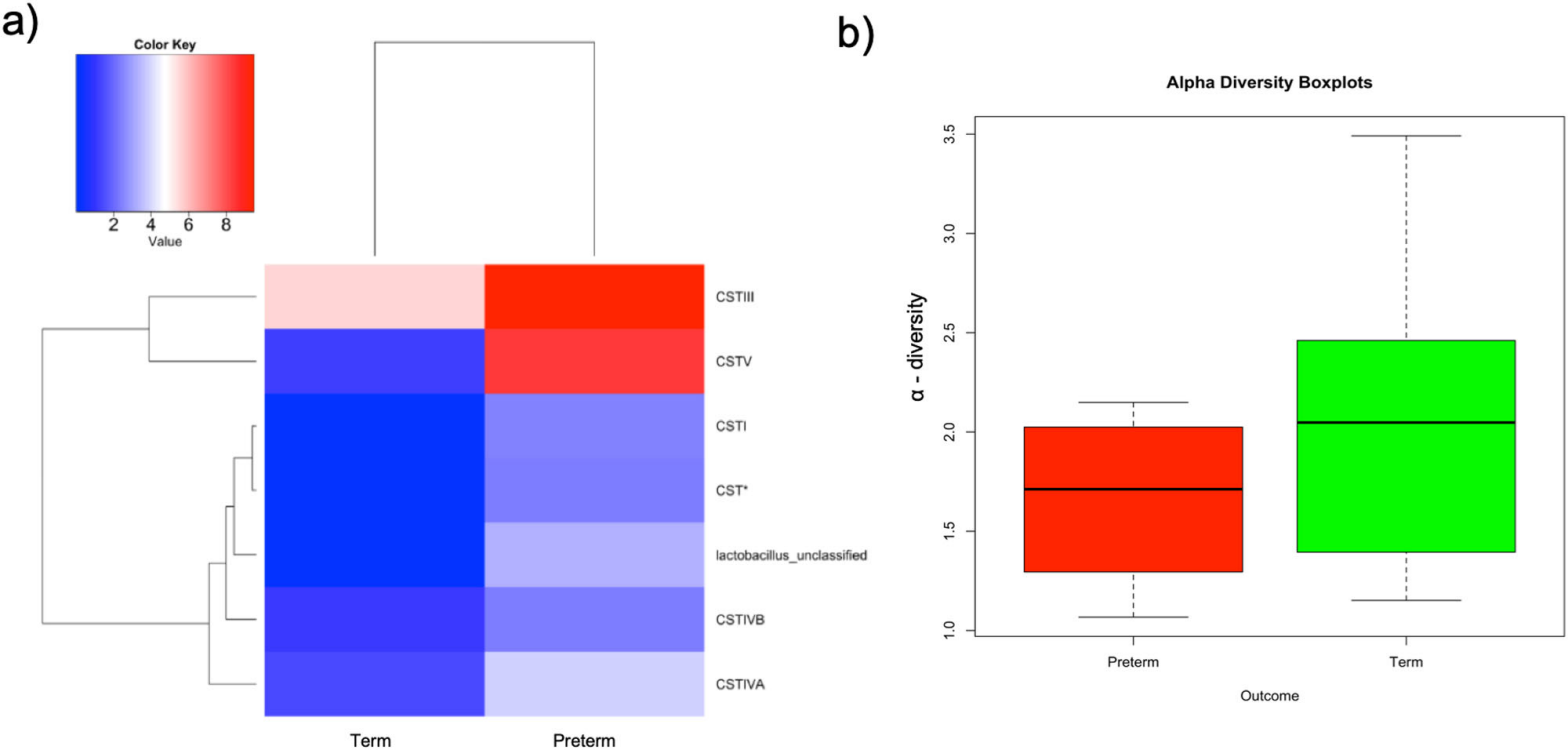
Changes in cervicovaginal bacterial community at gestational time point (GTP) 2 (26-28 weeks). **a** Community state types (CSTs) and **b** Alpha-diversity. *Key*: red = high or more dominant; blue = low or less dominant. Term = 13 vs. preterm = 6. Wilcoxon rank-sum test, *p* < 0.0001.

Similar to GTP1, the preterm-delivered women showed more CSTI, CSTIII, CSTV, CSTIVA, CSTIVB, CST* and *Lactobacillus*_unclassified compared to the term-delivered women at GTP2 (*p* < 0.0001) (Fig. 6a). The bacterial community diversity at GTP2 was also higher in the term- than preterm-delivered women (*p* < 0.0001) (Fig. 6b). Unlike GTP1, there were no significant differences in bacterial species abundance at GTP2. However, there were 14 and 17 significant positive correlations between the most abundant bacterial species in the term and preterm groups respectively. No negative correlation between the species was observed in any of the groups (Table 3).

**Fig. 7:**
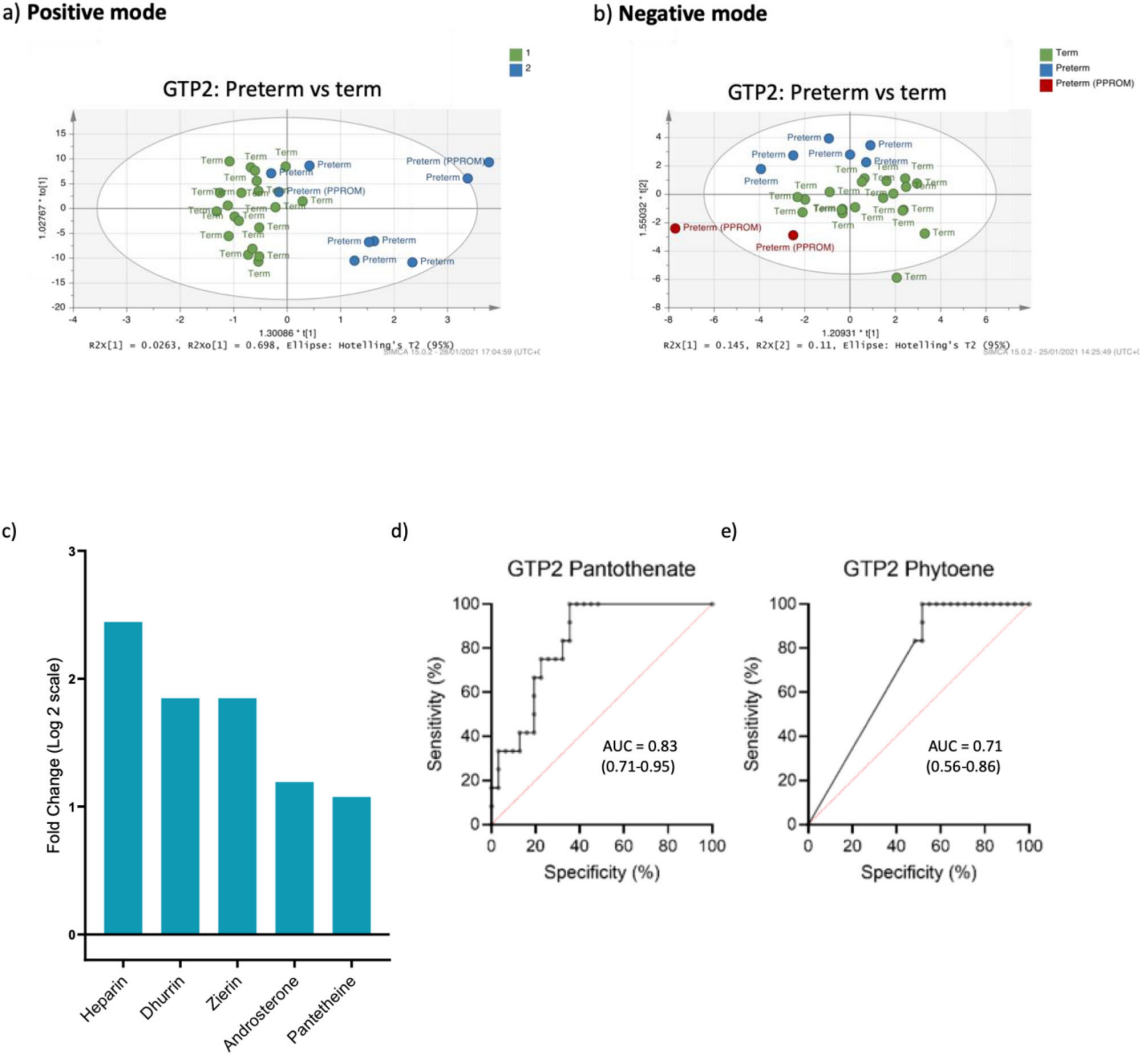
Orthogonal projections to latent structures discriminant analysis (OPLS-DA) of cervicovaginal fluid samples of preterm- and term-delivered women at gestational time point (GTP) 2 (26–28 weeks, *n* = 45). **a** Positive ionisation mode that detects peaks corresponding to protonated metabolites. **b** Negative ionisation mode, which detects peaks corresponding to deprotonated metabolites. **c** Fold change of metabolites that differ between preterm- vs. term-delivered women at GTP2. All the metabolites were upregulated in the women who delivered preterm compared to their term-delivered counterparts (Welch’s *t*-test). **d** and **e** Receiver operating characteristic curve analysis of the performance of pantothenate and phytoene for prediction of spontaneous preterm birth at GTP2 (Term = 31, Preterm = 14). *AUC* area under the ROC curve (95% confidence interval).

Furthermore, similar to GTP1, multivariate analysis of metabolite normalised % total ion count by OPLS-DA showed there was no clear separation of the samples between term- and preterm-delivered women at GTP2 in the positive and negative ionisation modes (Fig. 7a, b). However, 9 metabolites differed at GTP2 in term- vs. preterm-delivered women, but only 5 of these metabolites (heparin, pantetheine, androsterone, dhurrin and zierin) were significantly higher in the preterm- than the term-delivered women at GTP2 (Table 6, Fig. 7c) after the fold change (FC) cut-off of ±1 was applied. We also observed that pantothenate and phytoene were able to distinguish women at risk of sPTB at GTP2 (Table 7 and Fig. 7d, e).

**Table 6:** Fold change of cervicovaginal fluid metabolites that differ in preterm- vs. term-delivered women at GTP2 (26-28 weeks)

Metabolic pathway	Sub-pathway	Metabolite	Preterm/Term fold change (Log2)	*p*-value	KEGG ID	Function
Carbohydrate and carbohydrate conjugates	Disaccharide sulfates	Heparin	2.444	0.047	C00374	Anti-inflammatory agent^88^
Cyanogenic glycosides	Cyanoamino metabolism	Dhurrin	1.848	0.017	C05143	Inhibit cellular respiration^92^
		Zierin	1.848	0.017	C08345	
Lipids	Steroid hormone biosynthesis	Androsterone	1.192	0.044	C00523	• Masculinisation • Associated with PTB^93^
β amino acids and derivatives	Pantothenate and CoA biosynthesis; Carbapenem biosynthesis	Pantetheine	1.076	0.011	C00831	Oxidative stress and inflammation^89,91,120^

*p*-values are false discovery rate (FDR) adjusted.

*GTP* gestational time point, *KEGG* Kyoto Encyclopaedia of Genes and Genomes, *PTB* preterm birth.

**Table 7:** Predictive performance of cervicovaginal fluid metabolites for spontaneous preterm birth at second trimester

Metabolite	GTP	AUC (95% CI)	*p*-value
Pantothenate	GTP1	0.58 (0.41–0.74)	0.3891
	GTP2	0.83 (0.71–0.95)	**0.0009**
	Change from GTP1 to GTP2	0.71 (0.52–0.89)	**0.0317**
Phytoene	GTP1	0.62 (0.45–0.79)	0.1722
	GTP2	0.71 (0.56–0.86)	**0.0324**
	Change from GTP1 to GTP2	0.77 (0.63–0.92)	**0.0045**
Adenosine	GTP1	0.65 (0.48–0.81)	0.097
	GTP2	0.61 (0.41–0.80)	0.2728
	Change from GTP1 to GTP2	0.76 (0.62–0.91)	**0.0067**
Dehydrosafynol	GTP1	0.61 (0.44–0.78)	0.2114
	GTP2	0.69 (0.52–0.85)	0.058
	Change from GTP1 to GTP2	0.71 (0.55–0.88)	**0.0279**
Deoxyguanosine	GTP1	0.65 (0.48–0.81)	0.097
	GTP2	0.61 (0.41–0.80)	0.2728
	Change from GTP1 to GTP2	0.76 (0.62–0.91)	**0.0067**
Giganin	GTP1	0.65 (0.49–0.80)	0.097
	GTP2	0.6 (0.41–0.80)	0.291
	Change from GTP1 to GTP2	0.69 (0.51–0.87)	**0.0462**
Nonacosane	GTP1	0.63 (0.47–0.79)	0.1473
	GTP2	0.69 (0.49–0.88)	0.0617
	Change from GTP1 to GTP2	0.71 (0.53–0.89)	**0.0297**
Urate	GTP1	0.66 (0.49–0.83)	0.0616
	GTP2	0.69 (0.51–0.87)	0.0546
	Change from GTP1 to GTP2	0.71 (0.53–0.89)	**0.0279**

GTP1 (20–22 weeks, Term = 32, Preterm = 17); GTP2 (26–28 weeks, Term = 31, Preterm = 14); Change in abundance from GTP1 to GTP2 (Term = 31, Preterm = 12).

*AUC* area under receiver operating characteristic curve, *CI* confidence interval, *GTP* gestational time point.

*p* values that are statistically significant are shown in bold.

**Fig. 8:**
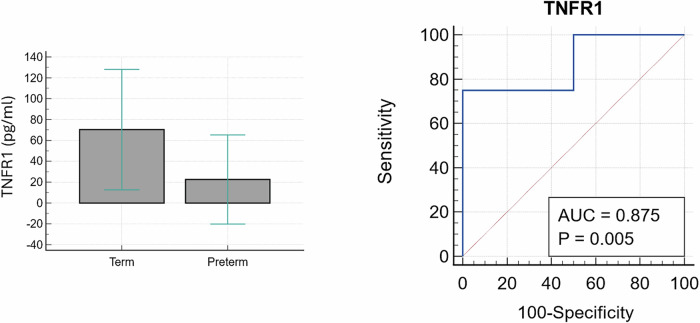
Receiver operating characteristic (ROC) curve analysis for TNFR1. TNFR1 was able to distinguish both groups at GTP2 (26–28 weeks) (*n* = 12, term = 8, preterm = 4). TNFR1 tumour necrosis factor receptor 1, AUC area under the ROC curve, GTP gestational time point.

For the chemokines/cytokines, only TNFR1 was significantly higher in term-delivered women compared to their preterm-delivered counterparts; and distinguished both groups with an AUC of 87.5% (*p* = 0.005) (Fig. 8 and Table 8).

**Table 8:** Predictive performance of significantly increased cervicovaginal fluid pro-inflammatory mediators and foetal fibronectin

Analyte	AUC (95%CI)	Sensitivity (%)	Specificity (%)	PPV (%)	NPV (%)	+LR	−LR	Criterion
TNFR1^a^	0.88 (0.56–0.99)	75.0	100.0	100.0	88.9	–	0.25	≤11.13
*GTP1/GTP2 ratio*								
CXCL10	0.68 (0.53–0.82)	42.86	93.6	75.0	78.4	6.64	0.61	>1.194
CXCL10 + FFN	0.74 (0.58–0.86)	64.29	79.31	60.0	82.1	3.11	0.45	>0.36

None of the detected chemokines/cytokines differed between term- vs. preterm-delivered women at GTP1.

*AUC* area under the ROC curve; Criterion (or cut-off), concentration that indicates the likelihood of spontaneous preterm birth. *CXCL10* C-X-C motif chemokine ligand 10 (CXCL10) or Interferon gamma-induced protein 10 (IP10), *FFN* foetal fibronectin, *GTP* gestational time point, *TNFR1* Tumour necrosis factor receptor 1, *NPV* negative predictive value, *PPV* positive predictive value, −*LR* negative likelihood ratio, +*LR* positive likelihood ratio.

^a^TNFR1 was the only analyte that differed significantly between term- vs. preterm-delivered women at GTP2.

### Changes in microbiota-metabolite and chemokine/cytokine profiles between GTP1 and GTP2

**Fig. 9:**
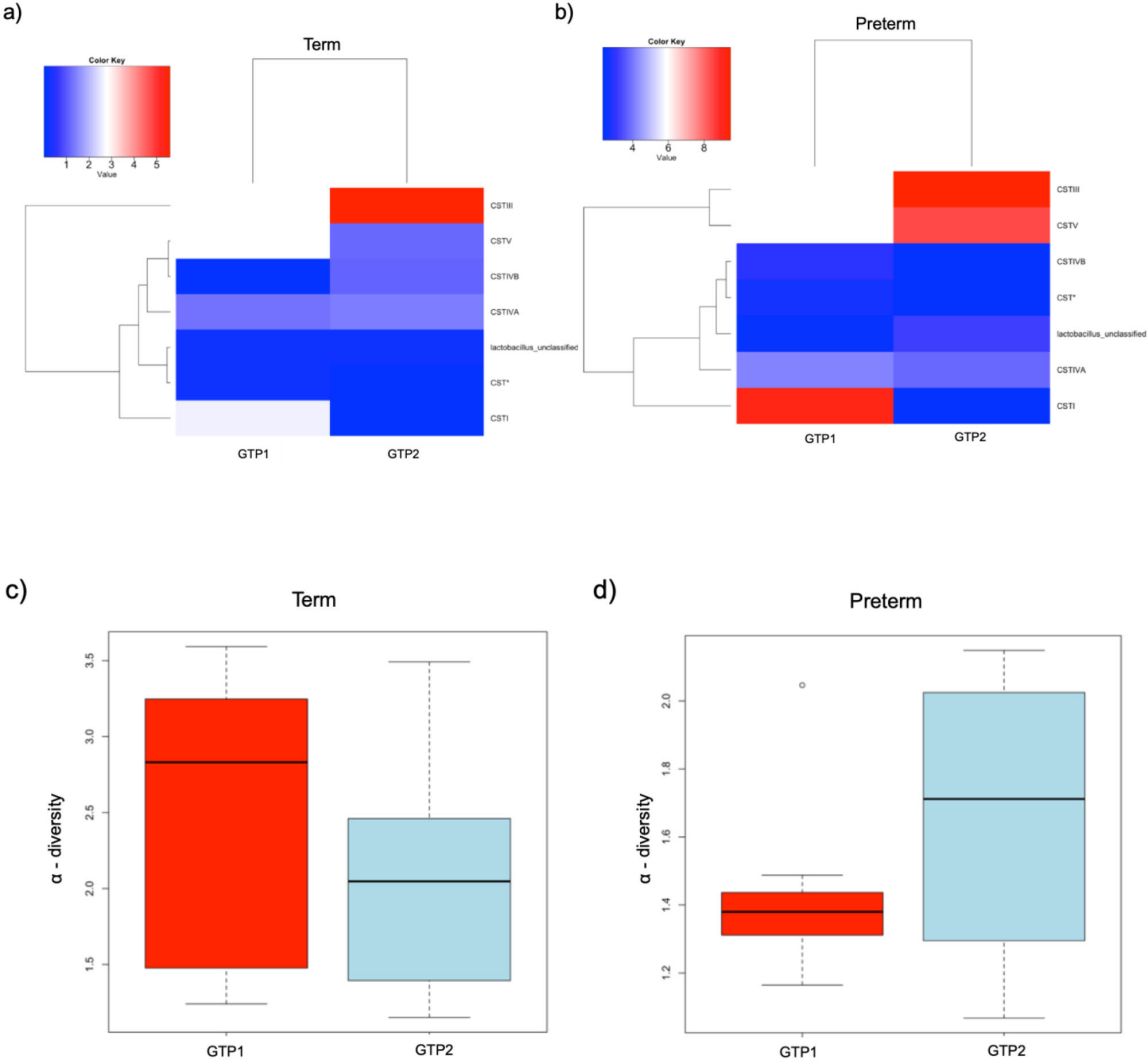
Changes in cervicovaginal bacterial community in asymptomatic pregnant women between gestational time points (GTPs). **a** and **b** Community state types (CSTs). *Key*: red = high or more dominant; blue = low or less dominant. **c** and **d** Alpha-diversity. There was a decrease in community diversity from GTP1 to GTP2 in the term-delivered women, while an opposite trend was observed in the preterm-delivered women (*p* < 0.0001). *GTP1*: 20–22 weeks (*n* = 21); *GTP2*: 26–28 weeks (*n* = 19), term = 26 and preterm = 14; Wilcoxon rank-sum test.

**Fig. 10:**
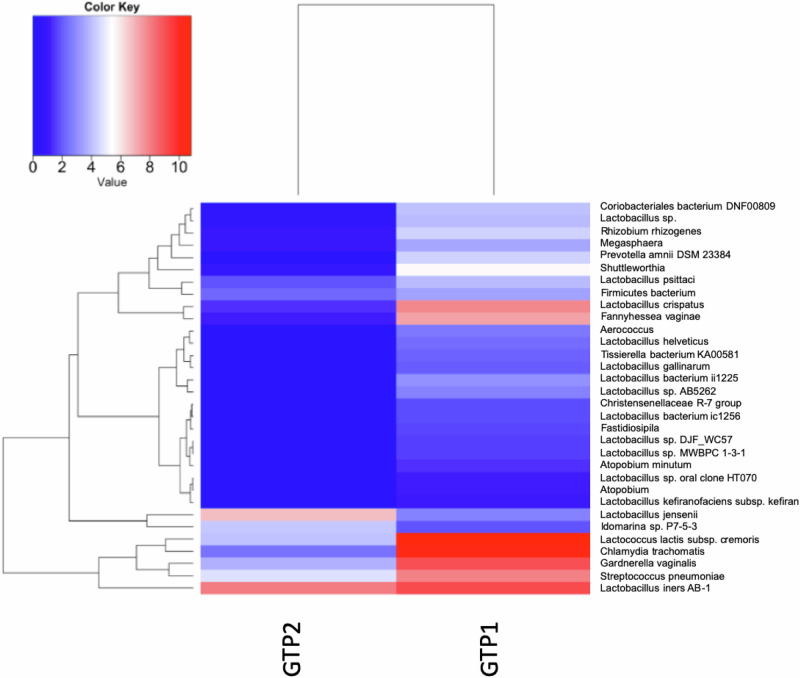
Comparison of bacterial species relative abundance between gestational time points. GTP1 (20–22 weeks, *n* = 21) vs. GTP2 (26–28 weeks, *n* = 19). Wilcoxon rank-sum test (*p* < 0.0001). GTP gestational time point.

In order to determine the temporal changes in vaginal microbiota metabolic activities and resultant inflammatory responses mediated by cytokines/chemokines^14,21–23^, we compared the bacterial compositions and concentrations of analytes between the two GTPs. In the women who delivered preterm, there was a decrease in CSTI, whereas CSTIII and CSTV increased. In the term-delivered women, only CSTIII increased, while CSTI and CSTV decreased. CSTIVA, CST* and *Lactobacillus*_unclassified did not change significantly (Fig. 9a, b). Within the groups, the alpha diversity increased from GTP1 to GTP2 in the women who delivered preterm, while it decreased from GTP1 to GTP2 in the women who delivered at term (Fig. 9c, d). A total of 40 CV fluid samples were analysed, and a comparison of the GTP1 (*n* = 21) vs. GTP2 (*n* = 19) samples showed 32 significantly different bacterial species. The GTP1 samples showed more diverse microbiota dominated by *L. iners*, *S. pneumoniae, G. vaginalis, C. trachomatis, F. vaginae, Shuttleworthia*, *P. amnii, Megasphaera, Coriobacteriales bacterium* DNF00809, including *L. crispatus*, and *Lactococcus lactis* subsp. *cremoris*. Whereas *L. jensenii* and *Idiomarina* sp. P7-5-3 were more dominant in the GTP2 samples (*p* < 0.0001, Fig. 10).

**Fig. 11:**
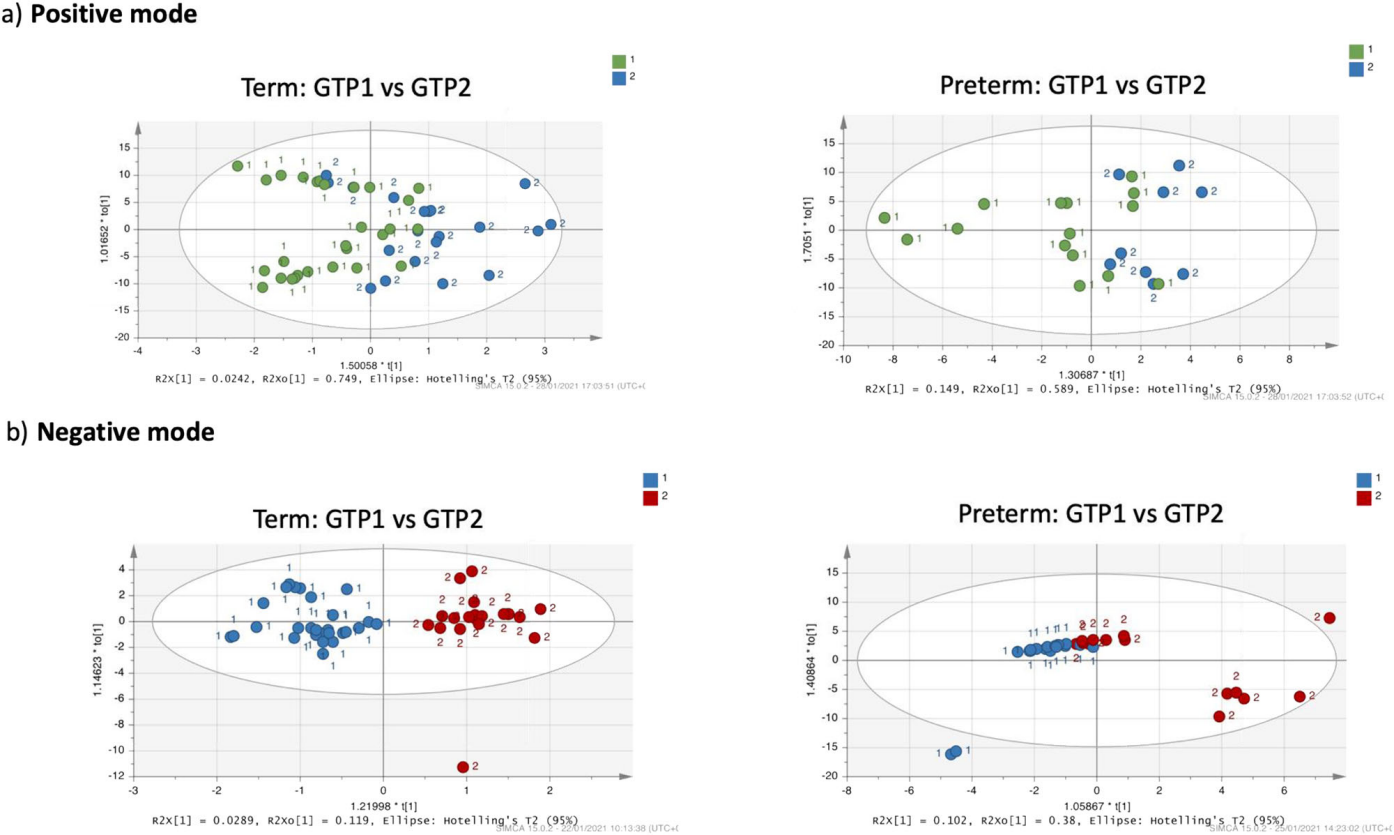
Orthogonal projections to latent structures discriminant analysis (OPLS-DA) of cervicovaginal fluid samples of preterm- and term-delivered women between gestational time points (GTPs) 1 and 2. **a** Positive ionisation mode that detects peaks corresponding to protonated metabolites. **b** Negative ionisation mode, which detects peaks corresponding to deprotonated metabolites. A clear separation was observed between GTP 1 and 2 in the term-delivered women (negative ionisation mode). Whereas, a similar but less distinct trend was observed in the preterm-delivered women perhaps due to the relatively smaller number of samples. GTP1 (*n* = 49) and GTP2 (*n* = 45).

**Fig. 12:**
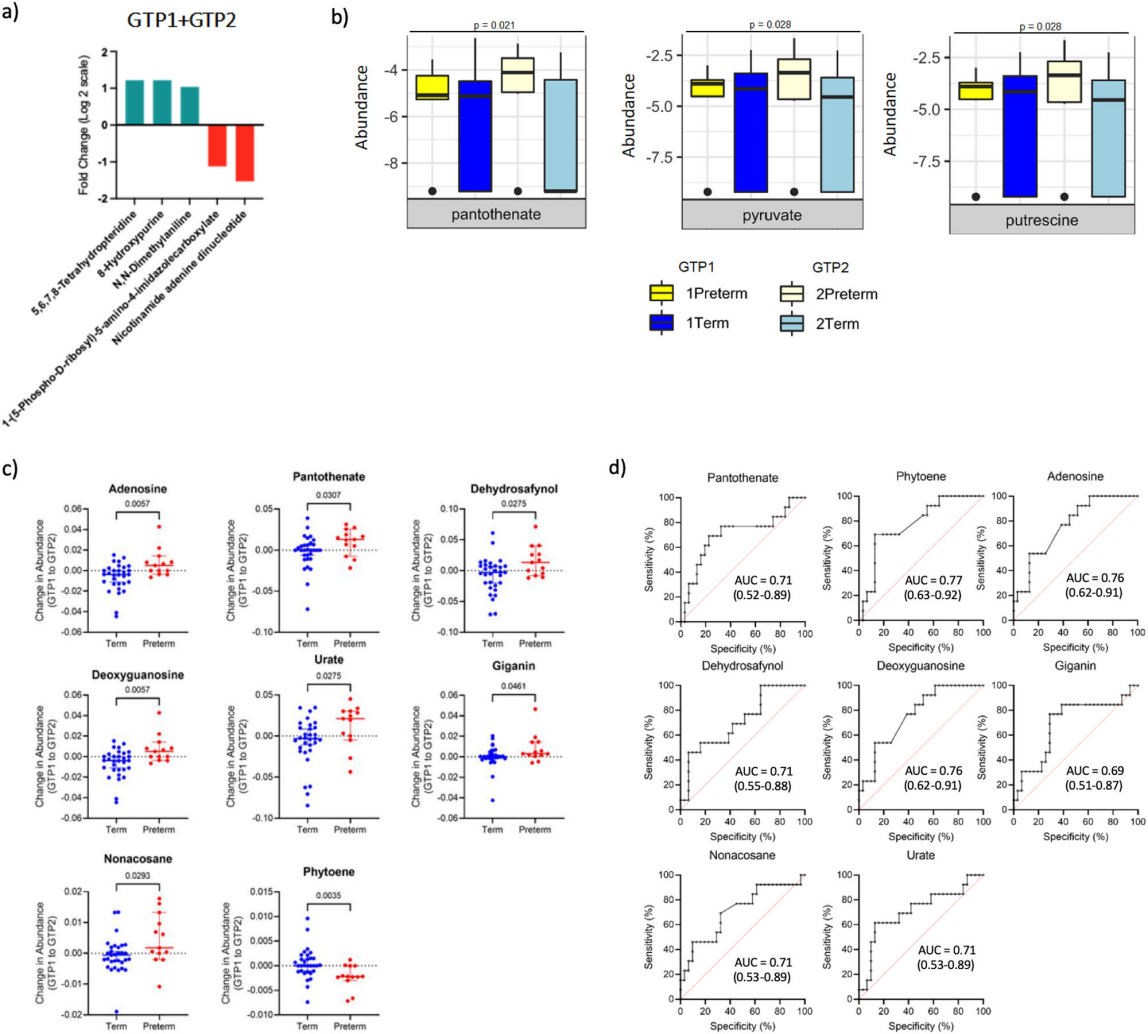
Comparison of cervicovaginal metabolite changes across gestation and in relation to birth outcome. **a** Fold change of metabolites that differ between GTP1 and GTP2 in the combined sample population. Nicotinamide adenine dinucleotide and 1-(5-Phospho-d-ribosyl)-5-amino-4-imidazolecarboxylate were upregulated in GTP1 samples and downregulated in GTP2 samples, while the other metabolites were upregulated in GTP2 samples. **b** Fold change in metabolite abundance from GTP1 to GTP2 in preterm- vs. term-delivered women determined by ANOVA. Pantothenate, putrescine, and pyruvate were significantly higher in the preterm-delivered women and increased from GTP1 to GTP2 in this group compared with term-delivered women. These metabolites also decreased significantly in the term-delivered women from GTP1 to GTP2 compared to women who delivered preterm. **c** Changes in metabolite abundance determined by subtracting the abundance at GTP1 from GTP2 (Welch’s *t*-test); and **d** these metabolite changes predicted risk of spontaneous preterm birth. GTP1 - 20-22 weeks (*n* = 49, term = 32, preterm = 17), GTP2—26–28 weeks (*n* = 45, term = 31, preterm = 14); GTP1 + GTP2 refers to combined sample population. Abundance = normalised % total ion count. *Midline* = median, error bars = 95% confidence interval; AUC area under receiver operating characteristic curve, GTP gestational time point.

Multivariate analysis of metabolite normalised % total ion count showed no clear separation of the samples between GTP1 and GTP2 in the positive ionisation mode. However, the negative ionisation mode indicated a clear separation between GTP1 and GTP2 in the term-delivered women, and a similar but less distinct trend was observed in the preterm-delivered women (Fig. 11). Fifty-four metabolites differed between GTP1 and GTP2. With the FC threshold of ±1 applied, only 5 metabolites including nicotinamide adenine dinucleotide (NAD), N,N-dimethylaniline, 8-hydroxypurine, 5,6,7,8-tetrahydropteridine, and 1-(5-Phospho-d-ribosyl)-5-amino-4-imidazolecarboxylate differed significantly between GTP1 and GTP2 (Table 9, Fig. 12a). Furthermore, 17 metabolites changed from GTP1 to GTP2 within and between the groups. These numbers reduced after the FC cut-off of ±1 was applied. Hence, only putrescine (*p* = 0.028), pyruvate (*p* = 0.028) and pantothenate (*p* = 0.021) were significantly higher in the preterm-delivered women and increased from GTP1 to GTP2 in this group compared to the term-delivered women. In contrast, these metabolites decreased significantly in the term-delivered women from GTP1 to GTP2 (Table 10 and Fig. 12b). We also observed that phytoene and isoliquiritigen were higher in the preterm-delivered women at GTP1 but decreased at GTP2 compared to the term-delivered women that experienced an increase. Other metabolites such as adenosine, dehydrostafynol, deoxyguanosine, jervine, nonacosane, thiamine, and urate were lower in the preterm-delivered women at GTP1 but became higher at GTP2 in these women compared to the term-delivered women. Giganin was higher in the term-delivered women at GTP1 but was lower at GTP2 than in the preterm-delivered women despite an increase from GTP1 to GTP2. Lanosterol and cycloartenol were higher in the preterm- than term-delivered women at GTP1 but decreased in both groups at GTP2; whereas phytofluene increased from GTP1 to GTP2 in the preterm-delivered women and was significantly higher in this group than the term-delivered women at GTP2 (Table 11 and Supplementary Fig. 3). When the metabolite changes were determined by subtracting the abundance at GTP1 from GTP2, there was a significant increase in pantothenate, adenosine, dehydrosafynol, deoxyguanosine, giganin, nonacosane and urate from GTP1 to GTP2 in the preterm-delivered women. Whereas, there was an increase in phytoene in the term-delivered women (Fig. 12c). These changes were indicative of sPTB (Table 7 and Fig. 12d).

**Table 9:** Fold change of cervicovaginal fluid metabolites that differ in all women between GTP1 and GTP2

Metabolic pathway	Sub-pathway	Metabolite	GTP2/GTP1 Fold change (Log2)	*p*-value	KEGG ID/HMDB ID	Function
Vitamins and cofactors	Biosynthesis of cofactors; Oxidative phosphorylation; Thiamine metabolism; Nicotinamide metabolism; AMPK signalling pathway; Aldosterone synthesis and secretion; Vitamin digestion and absorption; drug metabolism - other enzymes	Nicotinamide adenine dinucleotide	−1.5308708	0.026	C00003/HMDB0000902	Glycolysis and the citric acid cycle of cellular respiration
Pentose phosphates	Purine metabolism	1-(5-Phospho-d-ribosyl)-5-amino-4-imidazolecarboxylate	−1.122881	0.001	C04751/HMDB0006273	Inborn errors of metabolism
Amines	Dialkylarylamines	N,N-dimethylaniline	1.03769211	0.0003	C02846/HMDB0001020	
Purines and purine derivatives	Purinones	8-Hydroxypurine	1.21951229	0.013	NA/HMDB0012182	• Corticotropin-releasing hormone receptor antagonism, • Anti-rhinovirus activity, • Xanthine oxidase inhibiting activity • Benzodiazepine receptor binding
Pteridines	Pteridines and derivatives	5,6,7,8-tetrahydropteridine	1.21951229	0.013	C05650/HMDB0001216	• Cofactor for aromatic amino acids and ether lipids hydroxylation • Formation of nitric oxide (NO) from l-arginine • Biosynthesis of catecholamines • Autoxidation • Antioxidation

*p*-values are false discovery rate (FDR) adjusted.

*GTP* gestational time point: GTP1—20–22 weeks, GTP2—26–28 weeks, *HMDB* human metabolite database, *KEGG* Kyoto Encyclopaedia of Genes and Genomes.

**Table 10:** Metabolic pathways and sub-pathways of metabolites upregulated in preterm-delivered women and increased from GTP1 to GTP2 compared to term-delivered women

Metabolic pathway	Sub-pathway	Metabolite	Interaction *p*-value	Function
Vitamins and cofactors	Pantothenate and CoA biosynthesis; Vitamin digestion and absorption; β-alanine metabolism; Biosynthesis of cofactors	Pantothenate	0.021	Oxidative stress and inflammation^89–91^
Amines	Arginine and proline metabolism; d-amino acid metabolism; Tropane, piperidine and pyridine alkaloid biosynthesis; Biosynthesis of alkaloids derived from ornithine, lysine and nicotinic acid; Glutathione metabolism; Protein digestion and absorption; ABC transporters	Putrescine	0.028	• Associated with dysbiosis and BV • Cause the fishy smell of the vagina in BV^38,53^
Carbohydrates	Glycolysis; Citrate cycle; Phosphotransferase system (PTS); Biosynthesis of terpenoids and steroids; Biosynthesis of alkaloids derived from terpenoids and polyketide; Biosynthesis of phenylpropanoids; d-amino acid metabolism; Biosynthesis of alkaloids derived from histidine and purine; Microbial metabolism in diverse environments	Pyruvate	0.028	Intermediate for lactate or acetate production^38,53^

*p*-value obtained by two-way ANOVA.

*BV* bacterial vaginosis, *GTP* gestational time point: GTP1—20–22 weeks, GTP2—26–28 weeks.

**Table 11:** Cervicovaginal metabolite changes from GTP1 to GTP2 in preterm- and term-delivered women by ANOVA

Metabolite	Interaction *p*-value	KEGG or HMDB ID	Function
Nonacosane	0.008	C08384	• Component of pheromone • Chemical communication^121^
Adenosine	0.012	C00212	• Energy transfer • Signal transduction • Neurotransmitter • Vasodilator • Immunotoxin • Metabotoxin (HMDB0000050)
Deoxyguanosine	0.012	C00330	• Inherited metabolic disorder (Purine nucleoside phosphorylase (PNP) deficiency)^122^
Dehydrosafynol	0.014	C08447	Anit-inflammatory^123^
Cycloartenol	0.029	C01902	• Anti-inflammatory^124^
Lanosterol	0.029	C01724	• Animal and fungal sterol biosynthesis^125^
Phytoene	0.029	C05413	• Anti-oxidant • Anti-inflammatory^109,110^
Phytofluene	0.043	C05414	
Isoliquiritigen	0.037	HMDB0037318	• Enhance antitumor activity of cyclophosphamide^126^
Giganin	0.041	C08488	• Cytotoxic • inhibits mitochondrial electron transport systems^127^
Urate	0.022	C00366	• Anti-oxidant • Marker of PTB^102,103^
Jervine	0.049	C10811	• Anti-inflammatory, anti-tumour, anti-platelet, and anti-adipogenic agent^128,129^
Thiamine	0.049	C00378	• Vitamin B1 • Intracellular glucose metabolism • Antioxidant • Erythropoietic • cognition-and mood-modulator • Antiatherosclerotic • Putative ergogenic • Detoxification activities (HMDB0000235)

*p*-value obtained by two-way ANOVA.

*GTP* gestational time point: GTP1—20–22 weeks, GTP2—26–28 weeks, *HMDB* human metabolome database, *KEGG* Kyoto Encyclopaedia of Genes and Genomes, *PTB* preterm birth.

**Fig. 13:**
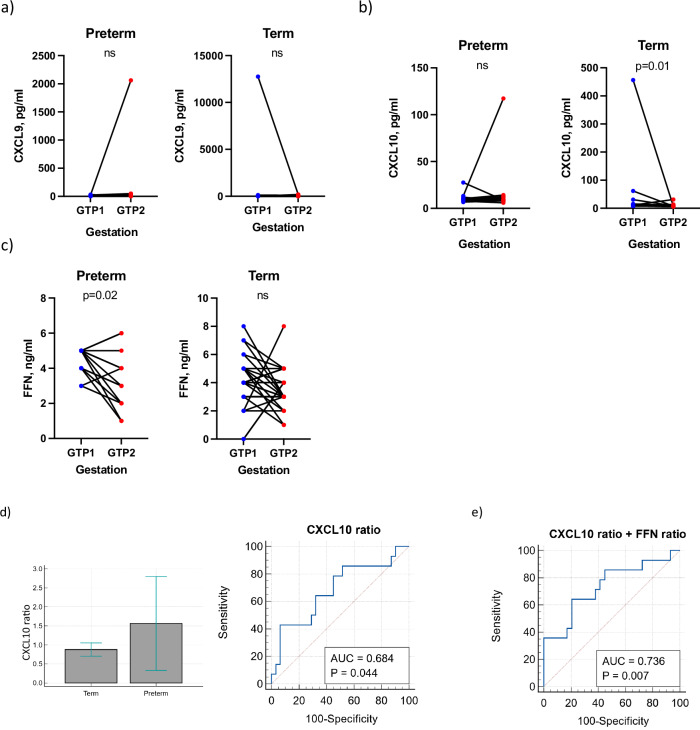
Gestational changes in cervicovaginal fluid levels of inflammatory markers between gestational time points. **a** CXCL9, **b** CXCL10 and **c** foetal fibronectin. (GTP1: 20–22 weeks, *n* = 45; and GTP2: 26–28 weeks, *n* = 45). CXCL9 and CXCL10 increased in the preterm-delivered women (though not significantly) from GTP1 to GTP2. By contrast, both chemokines decreased in the term women from GTP1 to GTP2. However, this trend was only significant for CXCL10 by 3-fold. Exclusion of the “outlier” data from the analysis did not change the outcome. Furthermore, FFN decreased with gestation irrespective of delivery outcome, especially in the preterm-delivered women (Wilcoxon matched-pairs signed rank test). **d** and **e** CXCL10 and a combination of CXCL10 and FFN levels expressed as ratios of GTP1/GTP2, distinguished the groups (*n* = 45, term = 31, preterm = 14). *CXCL9*, Chemokine (C-X-C motif) ligand 9 or monokine induced by gamma interferon (MIG); *CXCL10*, C-X-C motif chemokine ligand 10 (CXCL10) or Interferon gamma-induced protein 10 (IP10); FFN foetal fibronectin, AUC area under the ROC curve, GTP gestational time point.

Additionally, tracking the changes in CXCL9, CXCL10, and FFN in each woman from GTP1 to GTP2 according to delivery outcomes by Wilcoxon matched-pairs signed rank test showed a 3-fold decrease in CXCL10 in the term group from GTP1 to GTP2 (*p* = 0.01) (Fig. 13a, b). Exclusion of the “outlier” data from the analysis did not change the outcome. More so, irrespective of delivery outcome, FFN, which is an indicator of inflammation-induced disruption of the choriodecidual bond, decreased from GTP1 to GTP2, but this trend was only statistically significant in the women who delivered preterm (*p* = 0.02) (Fig. 13c). The FFN levels were generally lower than the normal range (<50 ng/mL) perhaps because the participants’ chances of delivery within 7–14 days of assessment/sampling was low. However, high levels of FFN in the CVF around 22 weeks’ gestation could be a sign of chronic inflammation (or cytokine)-mediated choriodecidual disruption, which may lead to sPTB^11,26,27,49,50^. Hence, we expressed CXCL9, CXCL10 and FFN as ratios of GTP1/GTP2 to track the changes of these analytes across the second trimester. We observed only the CXCL10 ratio was significantly higher in preterm-delivered women than term-delivered women, and was modestly indicative of sPTB (AUC = 68.4%). The combination of the ratios of CXCL10 and FFN by multiple regression increased the prediction of sPTB (AUC = 73.6%) (Table 8).

Furthermore, in the term and preterm groups, we attempted to perform correlations between the microbiome, metabolites and cytokines at GTP1 and GTP2 separately and combined. All the correlations were not statistically significant in the term group which had a relatively larger sample population. However, the same analyses were not successful in the preterm group due to the limited sample size.

## Discussion

In this cohort, women who experience sPTB show increasing concentrations of infectious, inflammation- and tissue damage-associated microbiota and metabolites across the second trimester. The immunological responses are characterised by an increase in chemokines associated with chronic inflammation of gestational tissues. We observed that in the *mid-second trimester* (20-22 weeks, GTP1), *F. vaginae, G. vaginalis, R. rhizogenes*, *C. bacterium*, *C. trachomatis* were more prevalent in the women who delivered preterm than in their term-delivered counterparts. These species, except *C. trachomatis* were also more abundant in the preterm-delivered women and corresponded with more CSTIV dominance in this group. Furthermore, *G. vaginalis* was significantly more abundant relative to either *L. crispatus* or *L. iners* in the women who delivered preterm; and *F. vaginae*, *G. vaginalis* and *G. vaginalis/L. iners* ratio identified women at risk of sPTB with high sensitivities, specificities and likelihood ratios. These microbiota changes were accompanied by upregulation of ten metabolites (except isobutrin) in the preterm-delivered women without a significant change in the detected proinflammatory mediators.

The presence of a high relative abundance of *F. vaginae, G. vaginalis, R. rhizogenes*, and *C. bacterium* was associated with a risk of sPTB. Low *L. crispatus* and *L. iners* relative to *G. vaginalis* were also associated with sPTB. These findings corroborate the view that an abundance of bacterial vaginosis-associated bacteria (BVAB) early in pregnancy may increase the risk of PTB^47,51^, particularly in high-risk populations^47^. However, there are instances where BVAB such as *Prevotella*, *Sneathia*, *Aerococcus* and *Coriobacteriaceae* family were decreased in women who delivered preterm, although they were Nugent-defined BV-positive African-American women sampled between 9 and 16 weeks of gestation^52^, in contrast to our predominantly BV-negative white population sampled at 20–22 and 26–28 weeks.

It is also interesting to observe that increased *L. crispatus* (relative to *G. vaginalis*), which is a hallmark of healthy vaginal microbiota^53^ and associated with term delivery^7^, was associated with term delivery in this study. This was accompanied by increased *L. iners* dominance in the term women relative to *G. vaginalis* which distinguished the term- from preterm-delivered women. This observation can be verified in future studies by ascertaining the quantitative frequencies of these key taxa by q-PCR^48^ instead of mere CST classification or relative abundance. More so, it is not just the relative abundance of the species but their functional capacities (metabolism) and eventual host immune response (production of inflammatory cytokines) that drive the ultimate pregnancy outcome^8–11,23,28,39,54,55^. Meanwhile, *L. iners* is the most prevalent bacterial species in the human vaginal microbiota^2,56,57^, which often coexists with *G. vaginalis* in high frequencies^48^, promotes vaginal health as a symbiont in some instances^58–60^, and relates inversely with PTB^61^. In other cases, it may promote dysbiosis and diseases such as BV^1,53,60^ as an opportunistic pathogen^58^ and PTB^62,63^. However, our results highlight the protective (eubiotic) characteristic of increased *L. iners* relative abundance. Our in vitro experiments show that *L. iners* does not produce significant amounts of BV- and PTB-associated metabolites, instead promotes eubiosis by producing significant amounts of L-lactate (*unpublished data*).

Interestingly, *L. gasseri* dominance was not observed in this cohort. This could be due to a mismatch within the primer. However, because *L. gasseri* was successfully amplified during optimisation of the primers, we suspect a possibly low to no abundance of *L. gasseri* in this population compared to our previous studies^7^, which detected *L. gasseri* dominance (CSTII) in 11% of 133 patients.

The upregulated metabolites in the preterm-delivered women possess anti-inflammatory and antioxidant properties, e.g. acacetin^64,65^, phloretin^66,67^, phytate^68–70^, xanthotoxin^71^, bergapten^72^, ostruthin^73,74^, gentamicin^75^; while others are found at sites of inflammation by nucleobase deamination e.g. xanthosine^76^, or promote inflammation, e.g. hydroxybupropion^77,78^, or associated with cancer dTMP^79,80^. Though their roles in the CV environment are yet to be fully established, their associations with inflammation at various body sites in humans and animal models are well documented. However, the identification of gentamicin (an antibiotic)^75^ indicates the possibility of contamination or recent exposure to the antibiotic. Isobutrin, which was the only upregulated metabolite in the women that delivered at term, is also an anti-inflammatory agent and inhibitor of PGE_2_ and matrix metalloproteinase (MMP)-1, 2, 9 and 10^81–83^. These anti-inflammatory, and PGE_2_ and MMPs inhibitory actions are important labour-related mechanisms that may have protected the women from sPTB and warrant further exploration. However, these metabolite changes were not accompanied by significant changes in the expression of the pro-inflammatory mediators we measured similar to our previous study^11^.

The chemical profile of the vaginal environment and associated CV fluid is widely regulated by the bacterial composition^8,9,28,38,42,84^, including the genome, transcriptome and proteome^85^. Consequently, we integrated the observed microbial CSTs, species relative abundances and differential expression of metabolites and pro-inflammatory mediators in those samples that have all data recorded. There were no significant correlations in the term-delivered women and similar analysis could not be conducted in the women who delivered preterm due to the very small sample numbers in this group. However, irrespective of delivery outcomes i.e., when the groups were combined together, there were significant correlations between CSTs, bacterial species, metabolites and chemokines/cytokines.

*By the late second trimester* (26–28 weeks, GTP2), the bacterial species abundance did not differ significantly between the groups. However, the preterm-delivered women showed more lactobacilli and mixed anaerobes-dominated CSTs, which were accompanied by upregulation of five metabolites. There was also a significant increase in TNFR1 in the term women which distinguished them from their preterm-delivered counterparts. These observations are somewhat similar to those of Romero et al. ^86^, which did not detect differences in the bacterial taxa, relative abundance and frequency of CSTs between term- and preterm-delivered patients, instead observed that vaginal microbiota changes with gestational age in women who deliver at term^86^. Another study reported higher within-sample variance in vaginal microbiome abundance in preterm-delivered women, with the most significant difference observed during the first trimester^87^. Again, the inconsistencies in microbiota data between studies and GTPs necessitated our investigation of the metabolic activities of the microbiota and eventual host immune response at each GTP. Consequently, we observed five upregulated metabolites in the women who delivered preterm, including the anti-inflammatory agent heparin^88^, pro-inflammatory pantetheine^89–91^, inhibitors of cellular respiration, i.e. dhurin and zierin^92^, and androsterone that was reportedly higher in amniotic fluid and placenta of preterm-delivered women^93^. The increased bacterial community diversity and downregulation of inflammatory and toxic metabolites in the term-delivered women were accompanied by a significant increase in TNFR1 in the same women, an observation that may have been influenced by the low number of samples in which TNFR1 was detected.

Hitherto, the implications of these metabolite changes for vaginal health and stimulation of inflammatory responses leading to sPTB are unclear. However, the upregulation of pantetheine (pro-inflammatory and oxidative stress mediator), dhurin, zeirin (inhibit cellular respiration), and androsterone (associated with PTB), in the women who delivered preterm versus heparin (an anti-inflammatory agent) may tilt the inflammatory response in favour of a pro-inflammatory state and oxidative damage of gestational tissues which promote sPTB^94–97^.

### How did GTP1 and GTP2 differ and what are the drivers?

As gestation progressed (from GTP1 to GTP2), the frequency of *L. crispatus*-dominated microbiota decreased, while the frequency of *L. iners*-dominated microbiota increased in both term- and preterm-delivered women. However, the frequency of *L. jensenii*-dominated microbiota increased in the preterm-delivered women, but decreased in the term-delivered women. There was also an increase in the bacterial community α-diversity in the preterm-delivered women, whereas the opposite trend was observed in the term-delivered women. These changes were accompanied by a significant increase in putrescine, pyruvate, and pantothenate in the preterm-delivered women, and a decline in the same metabolites in the term-delivered women across the same GTPs. Pantothenate and phytoene alone at GTP2, as well as the change of pantothenate, phytoene (increased in term-delivered women), urate, adenosine and others, which increased in preterm-delivered women from GTP1 to GTP2, were associated with risk of sPTB. In addition, women destined to deliver at term showed about a 3-fold decline in CXCL10 from GTP1 to GTP2. Whereas their preterm-delivered counterparts experienced a non-significant increase in both CXCL9 and CXCL10 across the same GTPs. Consequently, the GTP1/GTP2 ratio of CXCL10 was significantly higher in the women who delivered preterm and was indicative of sPTB with high PPVs and NPVs. This association with sPTB was improved when the ratio of foetal fibronectin across the same GTPs was added to the predictive model.

We analysed the changes in microbiota and immune response from GTP1 to GTP2 because the observations at each GTP may not provide a clear picture of the trends (drivers) that account for the differences in birth outcomes. Similar to previous studies^3^, the bacterial community α-diversity which was higher in the term-delivered women at GTP1, decreased to a less diverse community at GTP2. In contrast, the preterm-delivered women with initially lower diversity demonstrated an increase in α-diversity as gestation progressed to the late second trimester. This is consistent with previous reports that healthy pregnancy is associated with less carriage of BVAB, i.e. a community with high diversity^47,98,99^. However, it is at variance with a report of a cohort of predominantly African-American women who demonstrated stable community richness and diversity in term-delivered women, and decreasing richness and diversity in preterm-delivered women from first to second trimester^98^. Furthermore, both term- and preterm-delivered women had less frequency of *L. crispatus*-dominated and more frequency of *L. iners*-dominated microbiota at GTP2 compared to GTP1. However, the two groups differed in the frequency of *L. jensenii*-dominated microbiota, which was higher at GTP2 in the preterm-delivered women. Increased *L. jensenii* dominance is associated with the risk of sPTB^7^.

Moreover, pantothenate (vitamin B5), a product of pantetheine metabolism, was upregulated from GTP1 to GTP2 in women who delivered preterm but downregulated in women who had term deliveries. This change in pantothenate was also indicative of sPTB. By contrast, a previous study found pyridoxate (vitamin B6) to be downregulated in symptomatic women who delivered preterm^100^. Like pantothenate, we observed that the biogenic amine putrescine and pyruvate, which are usually associated with dysbiosis and BV^38,53^, were upregulated albeit not predictive of sPTB. Another biogenic amine (cadaverine) that collaborates with putrescine to cause the characteristic malodour of the vaginal fluid in patients with BV was found to significantly reduce the relative abundance of *Lactobacillus* spp. in vitro^101^. Whether putrescine exhibits such action is yet to be elucidated. We observed that the increasing trend of these dysbiotic and inflammatory metabolites in the preterm-delivered women from GTP1 to GTP2 corresponded with the increase in bacterial community diversity in the same women across the same GTPs. We also observed an increasing urate level in the preterm-delivered women from GTP1 to GTP2 that was higher than in their term-delivered counterparts at GTP2. Increased urate level was also indicative of sPTB. Higher maternal serum and salivary uric acid are associated with an increased risk of PTB in women with pre-eclampsia/eclampsia^102,103^. By contrast, phytoene decreased in the term-delivered women and distinguished women at risk of sPTB. Phytoene is included along with vitamin A in carotenoid biosynthesis. Since carotenoids are found in green leafy vegetables and yellow/orange fruits^104^, low phytoene in the women who delivered preterm may be linked to poor maternal nutrition. Although vitamin A or carotenoid supplementation is not recommended^104^, a 33% and 66% reduction in the prevalence of PTB and early PTB, respectively, were recorded in HIV-positive women who took Vitamin A^105^. Therefore, ultimately, it appears the observed delivery outcome is determined by the temporal metabolic (functional) profile of the vaginal microbiome. Subsequent studies can determine the expression levels of vanin-1/*VNN1* gene^91^ that encodes vanin-1 (pantetheinase) that metabolises pantetheine to pantothenate and cysteamine, which promotes oxidative stress and an inflammatory state^89–91^. Perhaps, cysteamine concentrations (and not pantetheine or pantothenate) would indicate the true state of inflammation in these cohorts/samples.

The temporal changes in the vaginal microbial and physicochemical structure can stimulate subclinical or asymptomatic inflammatory responses that may be of the chronic phenotype^31^. We observed that women destined to deliver at term showed ~3-fold decline in CXCL10, which is implicated in the pathophysiology of chronic placental inflammation^31^ across the second trimester. Whereas their preterm-delivered counterparts experienced a non-significant increase in both CXCL9 and CXCL10. Consequently, the GTP1/GTP2 ratio of CXCL10 was significantly higher in the women who delivered preterm and was indicative of sPTB that was improved by the addition of foetal fibronectin ratio. However, foetal fibronectin levels were generally low at each GTP and did not differ between the groups as the participant’s chances of delivery within 7–14 days of assessment/sampling were low. Although foetal fibronectin is not a cytokine, high levels of this glycoprotein in the CVF at/after 22 weeks gestation is a sign of inflammation (or cytokine)-induced disruption of the choriodecidual interface and increased risk of sPTB^11,26,27,49,50^. Therefore, CV fluid foetal fibronectin could be a proxy marker for chronic subclinical inflammation of gestational tissues such as the placenta and chorioamnion^49,50^.

### Key pathways and new insights on the vaginal host-microbial interactions

An insidious inflammatory response is often missed on routine clinical examination until symptoms of PTL begin to appear^106^. In this study, we observed vaginal bacterial community diversity, pantothenate, putrescine, pyruvate, and urate (markers of dysbiosis, inflammation, oxidative tissue damage and PTB), and CXCL10, a marker of chronic placental inflammation and apoptosis^47,107,108^ decreased from mid to late second trimester in women destined to deliver at term. Whereas phytoene, an antioxidant and anti-inflammatory agent^109,110^, increased in the same women across gestation. This resulted in a significantly higher CXCL10 ratio in the women who delivered preterm, distinguishing them from their term-delivered counterparts. These appear to be the first-time temporal changes in cervicovaginal CXCL10 and these metabolites will be determined in a cohort of predominantly white women and could indicate another explorable shift from association to mechanism in the pathophysiology of inflammation-induced sPTB. Change in CXCL10, pantetheine-pantothenate-cysteamine, putrescine, pyruvate, urate and phytoene could also be explored further as biomarkers of dysbiosis-inflammation-associated sPTB. That is, there is a possible microbial-metabolite-induced imbalance in CXCL10 between term- and preterm-delivered women as gestation progresses. We also showed that even though the vaginal microbiota was becoming less diverse and dominated by *L. crispatus* and *L. iners* as gestation progresses, an inflammation triggered in early gestation may persist and assume a chronic status mediated by rising CXCL10 levels. Though our cohort is dominated by white women, our findings have highlighted in part, the possible temporal dynamics of the complex host immune-microbiota interactions in pregnancy that determine the ultimate delivery outcome. This could also explain why antibiotic^51^ or probiotic^111^ treatments alone do not reduce PTB rates.

Despite its strengths and novelty, the current study could be improved with more women providing samples with all three datasets, i.e. microbiome, metabolites and cytokines. A sample collected at a third gestational time point e.g. 34–36 weeks could further enhance the coverage and assessment of temporal alterations in the vaginal microbiome and consequent immune response. Although a paired analysis of each participant between GTPs was performed for cytokines and FFN, our study could also benefit from a paired analysis of the microbiota changes from GTP1 to GTP2. However, this was not performed due to the low sample size in the subgroup analysed by 16S gene sequencing. Additionally, other subtypes of PTB (such as PTB < 34 weeks and PPROM) and sub-group (such as PTB in the Asian, African and Hispanic cohorts) analyses could not be conducted in the present study due to very low sample sizes of the other racio-ethnic groups. The generalisability of our findings could be enhanced by studying women from other racio-ethnic and geographical backgrounds, including African, Asian and Hispanic women as we have done in a South African cohort recently^112^. The undetectable cytokines could be analysed in smaller sample volumes by ELISA^112^ in place of cytometric bead array. We could not also include data from other gestational tissues such as the placenta and foetal membranes where the CXCR3 chemokines are usually detected in abundance during chorioamnionitis and other chronic inflammatory lesions^29,31,108^. Furthermore, though nanopore offers the advantage of long read sequencing reducing the challenge of accuracy and throughput, it is also prone to high mismatch and indel error rate and limited per read accuracy, which can be problematic when studying small or rare sequence differences^113,114^. We may have also missed some important metabolites due to suspending the CVF samples in PBS. This is because, in electrospray ionisation mode mass spectrometry, buffers and salts such as PBS can cause ion suppression and adduct formation in the mass spectrometer, thus, reducing signals and creating noisy spectra. They can also precipitate out and block the capillary tubing, therefore, the system needs more cleaning and maintenance^115^. For these reasons inorganic salts and buffers are, where possible, avoided.

The cervicovaginal microbiome and metabolome are dynamic and change across gestation, consistent with complex temporal interactions between the microbes and the host immune system that warrant further exploration. Characterising whether gestational changes in the cervicovaginal fluid profiles of microbial metabolites and cytokines associated with spontaneous preterm birth may enable pregnancy risk stratification.

## Supplementary information

Supplementary information

## Data Availability

The dataset has been deposited in the National Center for Biotechnology Information (NCBI) Sequence Read Archive (SRA) with BioProject accession number PRJNA836249. Other datasets used and/or analysed during the current study are available from the corresponding author upon reasonable request.
