# Supplementary material for: Vaginal host immune-microbiome-metabolite interactions associated with spontaneous preterm birth in a predominantly white cohort
Source: NPJ Biofilms Microbiomes. 2025 Mar 26;11:52. doi: 10.1038/s41522-025-00671-4 (PMC11947164; doi:10.1038/s41522-025-00671-4)
Supplement: Supplementary file 1 — Supplementary information [file 41522_2025_671_MOESM1_ESM.pdf]

Supplementary Table 1. Barcoded primer sequences

**Forward**	319F - CTCCTACGGGAGGCAGCAGT	**Reverse**	MCRevA - CTCACGACACGAGCTGACGAC
**Primer Name**	**Primer Sequence + Barcode**	**Primer Name**	**Primer Sequence + Barcode**
16S_319F_B<br>C01_F	AAGAAAGTTGTCGGTGTCTTTGTG<br>CTCCTACGGGAGGCAGCAGT	16S_MCRevA_<br>BC01_R	AAGAAAGTTGTCGGTGTCTTTGT<br>GCTCACGACACGAGCTGACGAC
16S_319F_B<br>C02_F	TCGATTCCGTTTGTAGTCGTCTGT<br>CTCCTACGGGAGGCAGCAGT	16S_MCRevA_<br>BC02_R	TCGATTCCGTTTGTAGTCGTCTG<br>TCTCACGACACGAGCTGACGAC
16S_319F_B<br>C03_F	GAGTCTTGTGTCCCAGTTACCAGG<br>CTCCTACGGGAGGCAGCAGT	16S_MCRevA_<br>BC03_R	GAGTCTTGTGTCCCAGTTACCAG<br>GCTCACGACACGAGCTGACGAC
16S_319F_B<br>C04_F	TTCGGATTCTATCGTGTTTCCCTA<br>CTCCTACGGGAGGCAGCAGT	16S_MCRevA_<br>BC04_R	TTCGGATTCTATCGTGTTTCCCT<br>ACTCACGACACGAGCTGACGAC
16S_319F_B<br>C05_F	CTTGTCCAGGGTTTGTGTAACCTT<br>CTCCTACGGGAGGCAGCAGT	16S_MCRevA_<br>BC05_R	CTTGTCCAGGGTTTGTGTAACCT<br>TCTCACGACACGAGCTGACGAC
16S_319F_B<br>C06_F	TTCTCGCAAAGGCAGAAAGTAGT<br>CCTCCTACGGGAGGCAGCAGT	16S_MCRevA_<br>BC06_R	TTCTCGCAAAGGCAGAAAGTAG<br>TCCTCACGACACGAGCTGACGA<br>C
16S_319F_B<br>C07_F	GTGTTACCGTGGGAATGAATCCTT<br>CTCCTACGGGAGGCAGCAGT	16S_MCRevA_<br>BC07_R	GTGTTACCGTGGGAATGAATCC<br>TTCTCACGACACGAGCTGACGA<br>C
16S_319F_B<br>C08_F	TTCAGGGAACAAACCAAGTTACG<br>TCTCCTACGGGAGGCAGCAGT	16S_MCRevA_<br>BC08_R	TTCAGGGAACAAACCAAGTTAC<br>GTCTCACGACACGAGCTGACGA<br>C
16S_319F_B<br>C09_F	AACTAGGCACAGCGAGTCTTGGTT<br>CTCCTACGGGAGGCAGCAGT	16S_MCRevA_<br>BC09_R	AACTAGGCACAGCGAGTCTTGG<br>TTCTCACGACACGAGCTGACGA<br>C
16S_319F_B<br>C10_F	AAGCGTTGAAACCTTTGTCCTCTC<br>CTCCTACGGGAGGCAGCAGT	16S_MCRevA_<br>BC10_R	AAGCGTTGAAACCTTTGTCCTCT<br>CCTCACGACACGAGCTGACGAC
16S_319F_B<br>C11_F	GTTTCATCTATCGGAGGGAATGGA<br>CTCCTACGGGAGGCAGCAGT	16S_MCRevA_<br>BC11_R	GTTTCATCTATCGGAGGGAATG<br>GACTCACGACACGAGCTGACGA<br>C
16S_319F_B<br>C12_F	CAGGTAGAAAGAAGCAGAATCGG<br>ACTCCTACGGGAGGCAGCAGT	16S_MCRevA_<br>BC12_R	CAGGTAGAAAGAAGCAGAATCG<br>GACTCACGACACGAGCTGACGA<br>C
16S_319F_B<br>C13_F	AGAACGACTTCCATACTCGTGTGA<br>CTCCTACGGGAGGCAGCAGT	16S_MCRevA_<br>BC13_R	AGAACGACTTCCATACTCGTGT<br>GACTCACGACACGAGCTGACGA<br>C
16S_319F_B<br>C14_F	AACGAGTCTCTTGGGACCCATAG<br>ACTCCTACGGGAGGCAGCAGT	16S_MCRevA_<br>BC14_R	AACGAGTCTCTTGGGACCCATA<br>GACTCACGACACGAGCTGACGA<br>C
16S_319F_B<br>C15_F	AGGTCTACCTCGCTAACACCACTG<br>CTCCTACGGGAGGCAGCAGT	16S_MCRevA_<br>BC15_R	AGGTCTACCTCGCTAACACCAC<br>TGCTCACGACACGAGCTGACGA<br>C
16S_319F_B	CGTCAACTGACAGTGGTTCGTACT	16S_MCRevA_	CGTCAACTGACAGTGGTTCGTA

C16_F	CTCCTACGGGAGGCAGCAGT	BC16_R	CTCTCACGACACGAGCTGACGAC
16S_319F_BC17_F	ACCCTCCAGGAAAGTACCTCTGATCTCCTACGGGAGGCAGCAGT	16S_MCRevA_BC17_R	ACCCTCCAGGAAAGTACCTCTGATCTCACGACACGAGCTGACGAC
16S_319F_BC18_F	CCAAACCCAACAACCTAGATAGGCCTCCTACGGGAGGCAGCAGT	16S_MCRevA_BC18_R	CCAAACCCAACAACCTAGATAGGCCTCACGACACGAGCTGACGAC
16S_319F_BC19_F	GTTCCTCGTGCAGTGTCAAGAGATCTCCTACGGGAGGCAGCAGT	16S_MCRevA_BC19_R	GTTCCTCGTGCAGTGTCAAGAGATCTCACGACACGAGCTGACGAC
16S_319F_BC20_F	TTGCGTCCTGTTACGAGAACTCATCTCCTACGGGAGGCAGCAGT	16S_MCRevA_BC20_R	TTGCGTCCTGTTACGAGAACTCATCTCACGACACGAGCTGACGAC
16S_319F_BC21_F	GAGCCTCTCATTGTCCGTTCTCTACTCCTACGGGAGGCAGCAGT	16S_MCRevA_BC21_R	GAGCCTCTCATTGTCCGTTCTCTACTCACGACACGAGCTGACGAC
16S_319F_BC22_F	ACCACTGCCATGTATCAAAGTACGCTCCTACGGGAGGCAGCAGT	16S_MCRevA_BC22_R	ACCACTGCCATGTATCAAAGTACGCTCACGACACGAGCTGACGAC
16S_319F_BC23_F	CTTACTACCCAGTGAACCTCCTCGCTCCTACGGGAGGCAGCAGT	16S_MCRevA_BC23_R	CTTACTACCCAGTGAACCTCCTCGCTCACGACACGAGCTGACGAC
16S_319F_BC24_F	GCATAGTTCTGCATGATGGGTTAGCTCCTACGGGAGGCAGCAGT	16S_MCRevA_BC24_R	GCATAGTTCTGCATGATGGGTTAGCTCACGACACGAGCTGACGAC
16S_319F_BC25_F	GTAAGTTGGGTATGCAACGCAATGCTCCTACGGGAGGCAGCAGT	16S_MCRevA_BC25_R	GTAAGTTGGGTATGCAACGCAATGCTCACGACACGAGCTGACGAC
16S_319F_BC26_F	CATACAGCGACTACGCATTCTCATCTCCTACGGGAGGCAGCAGT	16S_MCRevA_BC26_R	CATACAGCGACTACGCATTCTCATCTCACGACACGAGCTGACGAC
16S_319F_BC27_F	CGACGGTTAGATTCACCTCTTACACTCCTACGGGAGGCAGCAGT	16S_MCRevA_BC27_R	CGACGGTTAGATTCACCTCTTACACTCACGACACGAGCTGACGAC
16S_319F_BC28_F	TGAAACCTAAGAAGGCACCGTATCCTCCTACGGGAGGCAGCAGT	16S_MCRevA_BC28_R	TGAAACCTAAGAAGGCACCGTATCCTCACGACACGAGCTGACGAC
16S_319F_BC29_F	CTAGACACCTTGGGTTGACAGACCCTCCTACGGGAGGCAGCAGT	16S_MCRevA_BC29_R	CTAGACACCTTGGGTTGACAGACCCTCACGACACGAGCTGACGAC
16S_319F_BC30_F	TCAGTGAGGATCTACTTCGACCCACTCCTACGGGAGGCAGCAGT	16S_MCRevA_BC30_R	TCAGTGAGGATCTACTTCGACCCACTCACGACACGAGCTGACGAC
16S_319F_BC31_F	TGCGTACAGCAATCAGTTACATTGCTCCTACGGGAGGCAGCAGT	16S_MCRevA_BC31_R	TGCGTACAGCAATCAGTTACATTGCTCACGACACGAGCTGACGAC
16S_319F_BC32_F	CCAGTAGAAGTCCGACAACGTCATCTCCTACGGGAGGCAGCAGT	16S_MCRevA_BC32_R	CCAGTAGAAGTCCGACAACGTCATCTCACGACACGAGCTGACGAC

16S_319F_BC33_F	CAGACTTGGTACGGTTGGGTAACTCTCCTACGGGAGGCAGCAGT	16S_MCRevA_BC33_R	CAGACTTGGTACGGTTGGGTAACTCTCACGACACGAGCTGACGAC

Supplementary Note 1

Multiplexed bead-based immunoassay (Cytometric Bead Array)
Preparation of the standards
The BD CBA Human Enhanced Sensitivity Flex Set standards were prepared by pooling all lyophilized standard spheres into a 15 mL polypropylene tube, reconstituting with 4 mL of Assay Diluent and allowing the mixture to equilibrate at room temperature for 15 minutes. Ten 12 x 75 mm tubes were labelled and arranged in the following order: 0, 10, 20, 40, 80, 156, 312, 625, 1250 and 2500 (Top Standard). Then, 460 µL of Assay Diluent was transferred into the Top Standard tube without the reconstituted standard. 500 µL of Assay Diluent was transferred into the other nine 12 x 75 mm tubes. 25 µL of the reconstituted standards was transferred into the Top Standard tube and mixed by gentle vortexing for about 3 seconds. Serial dilution was performed by transferring 200 µL from the Top Standard tube into the dilution tube labelled 10, continuing serially up to the 1250 labelled dilution tube. The tube labelled as 0 containing only Assay Diluent was prepared as the 0 fg/mL negative control.
Mixing the cytokine-specific capture beads in solution
After diluting (thoroughly resuspending) the capture beads to their optimal concentration according to the number of tests in the experiment as indicated below, 25 µL of the capture bead solution was transferred into each tube of both unknown samples and standards. The tubes were gently mixed and incubated for one hour at room temperature. The incubation was performed in the absence of ultraviolet light, in order for the capture antibodies to adequately bind to the cytokines contained in each sample and standard.
Total volume of diluted beads needed:
96 samples in total (GTP n=51, GTP n=45)+10 standard samples =106. We tested 120 samples to avoid pipetting errors.
25 µL of diluted capture bead mix into each tube of both samples and standards, thus:
25 x 120 = 3000 µL total volume of beads
*We have 6 beads, one for each cytokine (*CXCL9, CXCL10, CXCL11, TNF-α, Eotaxin and TNFR1) *and 0.5 µL for each bead per test sample.*

0.5 x 120 = 60 µL
6 beads x 60 = 360 µL volume of each beads tested
Volume of Capture Bead Diluent = 3000 - 360 = 2640 µL

Preparing Detection Reagents (Parts A and B)
During the incubation time, Detection Reagent diluent provided with each BD CBA Human Enhanced Sensitivity Flex Set was prepared by calculating the total volume of the Detection Reagent (Part A) needed for the experiment, and subtracting the volume needed for each Human Detection Reagent to obtain the volume of the Detection Reagent diluent as indicated below:
Volume of Detection Reagent diluent needed to dilute the Detection Reagent (Part A) = Total volume of diluted Detection Reagent required - volume needed for each Detection Reagent
25 µL of the Mixed Human Detection Reagents (i.e. mixture of detection reagent (Part A) and diluent) were added into each sample tube and incubated further for another 1½ hour protected from light at room temperature to allow organometallic (sandwich) complexes to be formed.
The lyophilized Enhanced Sensitivity Detection Reagent (Part B) provided in the Human Enhanced Sensitivity Master Buffer Kit was prepared by reconstitution with 550 µL of the Detection Reagent diluent and incubated for 15 minutes at room temperature, away from light. 4.5 mL of the Detection Reagent diluent was transferred to a 15 mL conical polypropylene tube, followed by the transfer of 0.5 mL of the reconstituted Enhanced Sensitivity Detection Reagent (Part B) and mixed by gentle vortexing.
Assay procedure
Finally, the Human Enhanced Sensitivity Flex Set Assay was performed. The filter plate was washed with 100 µL of Wash Buffer and aspirated until drained. 50 µL of the standard dilutions were transferred to the first eight tubes, followed by the transfer of 25 µL of the unknown samples to the following tubes. 25 µL of capture beads were added into each assay tube, followed by 25 µL of the mixed Human Detection Reagent (Part A) and 100 µL of the Enhanced Sensitivity Detection Reagent (Part B). The tubes were incubated for 1 hour at room temperature

and centrifuged at 200 x *g* for 5 minutes, after adding 1 mL of Wash Buffer. The supernatant was then carefully aspirated and discarded safely.

The FCAP Array v1.0 software was used to generate mean fluorescence intensity (MFI) for both standards and CV fluid samples. A calibration curve was created with the fluorescence data (MFI) collected from the standard samples using the *RIA or ELISA - Interpolate unknowns from sigmoidal curve* function of GraphPad prism. Cytokine concentration in the samples were subsequently extrapolated from the standard curve. Uniformity of data was maintained by using the same procedure for each cytokine measurement, operated once with the same equipment and by the same operator.

Supplementary Note 2

Correlation of microbiota, metabolite and chemokine/cytokine profiles at GTP1

At GTP1, 14 CV fluid samples had both microbiota and metabolome data and we observed some correlations between the CSTs and most abundant metabolites (Supplementary Figure 1A). However, only the strongest correlations are reported in the subsequent texts. CSTI, CST* and *Lactobacillus*_unclassified correlated positively with acacetin and xanthosine, but negatively with isobutrin and phytate ($p < 0.05$). In contrast, CSTIII, CSTIVA and CSTIVB correlated positively with isobutrin, but negatively with ostruthin, hydroxybupropion, gentamicin, xanthotoxin and bergapten ($p < 0.05$). CSTIVB was also negatively correlated with phloretin ($p < 0.05$) (Supplementary Figure 1A). Furthermore, there were positive correlations between acacetin and *Lactobacillus* species (e.g. *L. crispatus, L. acidophilus* etc.), *Lactococcus lactis* subsp. *cremoris* and *C. trachomatis*; while it correlated negatively with *L. iners* AB-1, *F. vaginae* and *Megasphaera*. Xanthosine also correlated with *Lactobacillus* sp., *C. trachomatis, S. pneumoniae* and *R. rhizogenes* positively; and negatively with *F. vaginae* and *Megasphaera*. Isobutrin correlated positively with *G. vaginalis, F. vaginae, Shuttleworthia, Megasphaera, L. iners* AB-1, *Idiomarina* sp. P7-5-3 and *Lactobacillus* sp. AB5262. It was negatively correlated with *L. psittaci, L. helveticus, L. crispatus, L. acidophilus, L. gallinarum* and *C. trachomatis*. There were also weak positive correlations between phloretin and *S. pneumoniae, R. rhizogenes, L. iners* AB-1, *Lactobacillus* sp. G3_1_2M02; which correlated negatively with *F. vaginae, Shuttleworthia, Megasphaera* and *Idiomarina* sp. P7-5-3. Though weakly, phytate correlated positively with *Shuttleworthia* and *G. vaginalis*, and negatively with *L. crispatus* and other *Lactobacillus* sp. Furthermore, there were negative correlations between xanthotoxin and *Idiomarina* sp. P7-5-3, *G. vaginalis, L. iners* AB-1, *F. vaginae, Megasphaera* and *S. pneumoniae*; and weak positive correlations with *Shuttleworthia, C. trachomatis* and *Lactobacillus* sp. including *L. crispatus* (Supplementary Figure 1C).

With an adjusted FC threshold of ±0.5, the chemokines/cytokines showed some significant correlations with the microbiota and only the strongest correlations were emphasised (Supplementary Figure 2). That is, at GTP1, CSTI and CSTIII correlated negatively with CXCL9, CXCL10 and TNFR1, whereas CSTIVB and to a lesser extent CSTIVA and CST* correlated positively with all 3 inflammatory mediators (Supplementary Figure 2A). Similar to the correlations with CSTs (Supplementary Figure 2C), CXCL9, CXCL10 and TNFR1 positively correlated with *Shuttleworthia* only; and negatively with the *Lactobacillus* species, *L. lactis*, and other anaerobes such as *G. vaginalis, F. vaginae, Megasphaera, S. pneumoniae, R. rhizogenes, Idiomarina* sp. P7-5-3 and *C. trachomatis*. The inflammatory mediators also showed mild negative correlation with *S. sanguinegens, Dialister, Aerococcus, Parvimonas Coriobacteriales bacterium* DNF00809, *Clostridiales bacterium* KA00274 and *Streptomyces rameus*. There were mild positive correlations between CXCL10, TNFR1 and isobutrin; and between CXCL9, CXCL10 and ostruthin. The chemokines/cytokines correlated negatively with the other metabolites (Supplementary Figure 2E).

Correlation of microbiota, metabolite and chemokine/cytokine profiles at GTP2

CSTI correlated positively with dhurrin and zierin, and negatively with heparin and androsterone ($p < 0.05$) (Supplementary Figure 2B). While CSTV correlated positively with pantetheine and negatively with androsterone, dhurrin, zierin and heparin ($p < 0.05$). CST* correlated positively with heparin and to a lesser extent with androsterone, dhurrin, zierin and pantetheine ($p < 0.05$). CSTIVA negatively correlated with heparin, pantetheine and androsterone ($p < 0.05$) (Supplementary Figure 1B). There were notable positive correlations between pantetheine and *L. jensenii*, *Idiomarina* sp. P7-5-3, *L. acidophilus*, *L. crispatus*; and negative correlation with *Bifidobacterium*. The cyanogenic glycosides i.e. dhurrin and zierin correlated positively with *Coriobacteriales bacterium* DNF00809, *C. trachomatis*, *L. acidophilus*, *L. crispatus*, *R. rhizogenes*; and negatively with *Bifidobacterium, S. pneumoniae* and *L. lactis* subsp. *cremoris*. Heparin strongly correlated with *L. acidophilus* positively. There were positive correlations between androsterone and *Idiomarina* sp. P7-5-3 and *L. acidophilus*; and negative correlations with *L. jensenii*, *L. lactis* subsp. *cremoris, S. pneumoniae, R. rhizogenes* and *C. trachomatis* (Supplementary Figure 1D). CSTI correlated positively with CXCL10 and negatively with CXCL9, and TNFR1; whereas CST* correlated positively with TNFR1 and negatively with CXCL10. CSTIVA and CSTIVB also correlated negatively with TNFR1 and CXCL9. CSTIII correlated negatively with all three inflammatory mediators; whereas CSTV correlated negatively with CXCL9 and TNFR1 and mildly positively with CXCL10 (Supplementary Figure 2B). CXCL10 showed a strong negative correlation with *L. acidophilus*,

and mild positive correlations with *C. trachomatis*, *Coriobacteriales bacterium* DNF00809, *Idiomarina* sp. P7-5-3, *L. crispatus, L. jensenii, L. psittaci* and *R. rhizogenes*. CXCL10 also had mild negative correlations with *L. iners* AB-1, *L. lactis, S. pneumoniae, Finegoldia* and *Bifidobacterium*. By contrast to CXCL10, TNFRI had a strong positive correlation with *L. acidophilus* and mildly with *L. psittaci*, while CXCL9 only had a mild positive correlation with *L. psittaci* (Supplementary Figure 2D). CXCL9 and CXCL10 showed strong positive correlation with androsterone and weakly correlated with pantetheine. On the other hand, TNFR1 showed strong negative correlation with dhurrin, pantetheine and zierin ($p < 0.05$) (Supplementary Figure 2F).

Correlation of microbiota, metabolite and chemokine/cytokine profiles GTP1 vs. GTP2

A total of 28 CV fluid samples (GTP1 = 14 vs. GTP2 = 14) had both microbiota and metabolome data. We observed some correlations between the CSTs and most abundant metabolites. However, only the strongest correlations are reported in the subsequent texts. In the combined sample population, 5,6,7,8-tetrahydropteridine and 8-hydroxypurine correlated positively with CSTIII and negatively with CSTI, CST* and CSTV. CSTV was also negatively correlated with 1-5-phospho-D-ribosyl-5-amino-4-imidazolecarboxylate. There were also significant correlations between the dominant bacterial species and metabolite. Notably, *Lactobacillus* sp. AB5262, *F. vaginae*, *A. minutum* and to a lesser extent *L. iners* AB-1, *Idiomarina* sp. P7-5-3, *G. vaginalis* and *Firmicutes bacterium* positively correlated with 5,6,7,8-tetrahydropteridine and 8-Hydroxypurine; and negatively with *L. crispatus* and *L. jensenii*. Both *L. crispatus* and *L. jensenii* also negatively correlated with NAD and 1-(5-Phospho-D-ribosyl)-5-amino-4-imidazolecarboxylate. Furthermore, *Shuttleworthia* and to a lesser extent *Aerococcus*, *Lactobacillus* sp. AB5262 and *Lactobacillus* sp. MWBPC 1-3-1 correlated positively with N,N-dimethylaniline. In the combined sample population (n = 93), CSTIII, CSTIVB and to a lesser extent CSTIVA correlated positively with CXCL9 and CXCL10, while CSTI, CSTIII, CSTV and CST* correlated negatively with TNFR1.

The chemokines/cytokines showed some significant correlations with the bacterial species when both GTP1 (n = 14) and GTP2 (n = 14) samples were combined. CXCL10 strongly correlated positively with *Shuttleworthia* and weakly with *L. iners* AB-1, *S. pneumoniae* and *Firmicutes bacterium*. CXCL9 showed similar positive correlations with the bacterial species. Both CXCL9 and CXCL10 correlated negatively with *L. crispatus, L. jensenii, L. helveticus, L. psittaci, L. lactis* subsp. *cremoris, G. vaginalis, C. trachomatis, F. vaginae, A. minutum, Megasphaera, Aerococcus* and *Idiomarina* sp. P7-5-3. TNFR1 was only mildly positively correlated with *Shuttleworthia* and *R. rhizogenes*; but correlated negatively with *L. iners* AB-1, *L. crispatus, L. jensenii, L. helveticus, L. psittaci, L. lactis* subsp. *cremoris, G. vaginalis, C. trachomatis, F. vaginae, A. minutum, Megasphaera, Idiomarina* sp. P7-5-3, *Coriobacteriales bacterium* DNF00809, *Tissierella bacterium*, *Aerococcus* and *Firmicutes bacterium*. In the combined sample population, only TNFR1 and NAD correlated positively.

a)

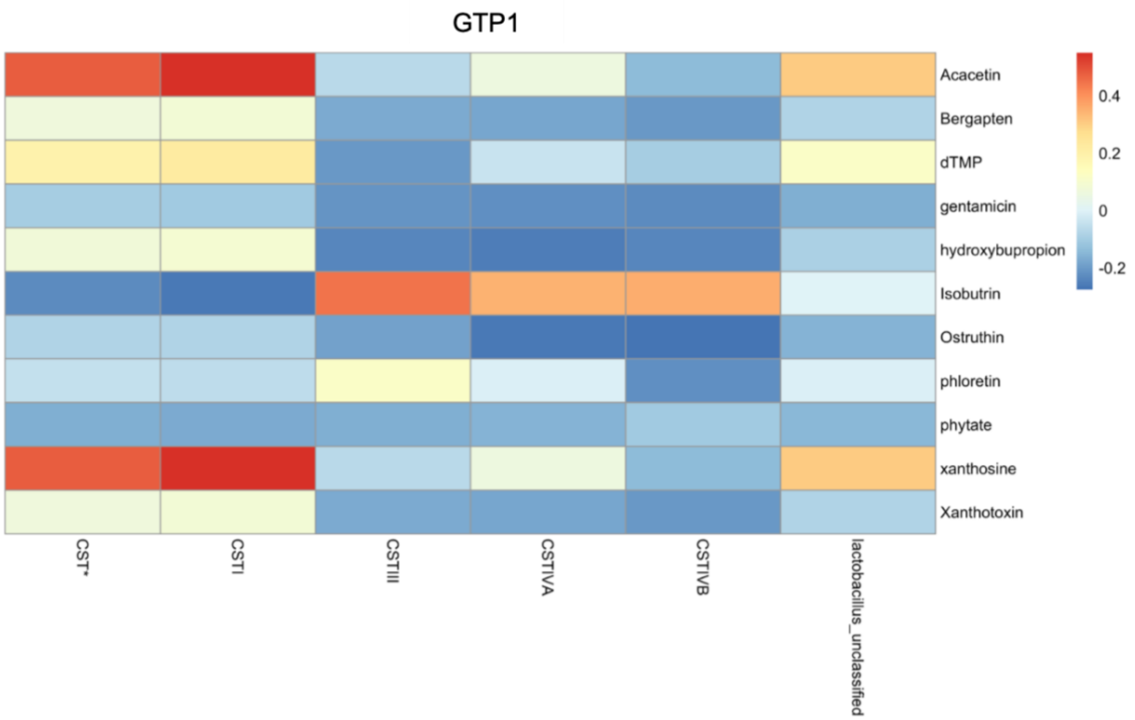
GTP1
Acacetin
Bergapten
dTMP
gentamicin
hydroxybupropion
Isobutrin
Ostruthin
phloretin
phytate
xanthosine
Xanthotoxin
0.4
0.2
0
-0.2
CST*
CSTI
CSTIII
CSTIVA
CSTIVB
lactobacillus_unclassified

b)

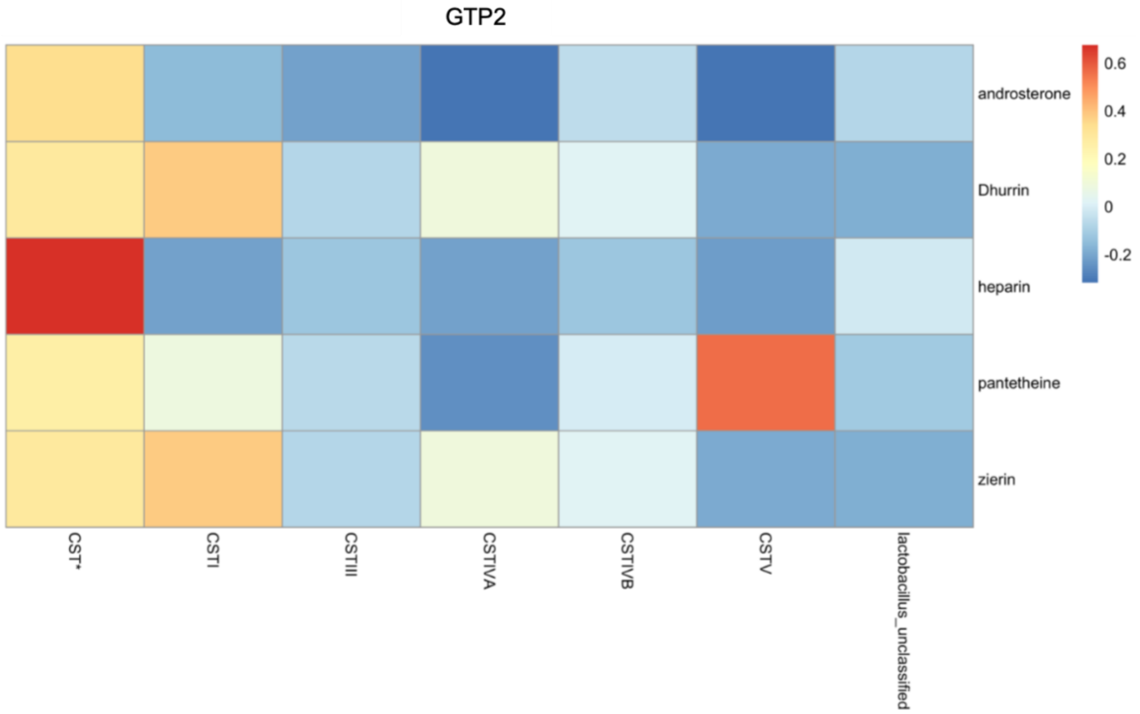
GTP2
androsterone
Dhurrin
heparin
pantetheine
zierin
0.6
0.4
0.2
0
-0.2
CST*
CSTI
CSTIII
CSTIVA
CSTIVB
CSTV
lactobacillus_unclassified

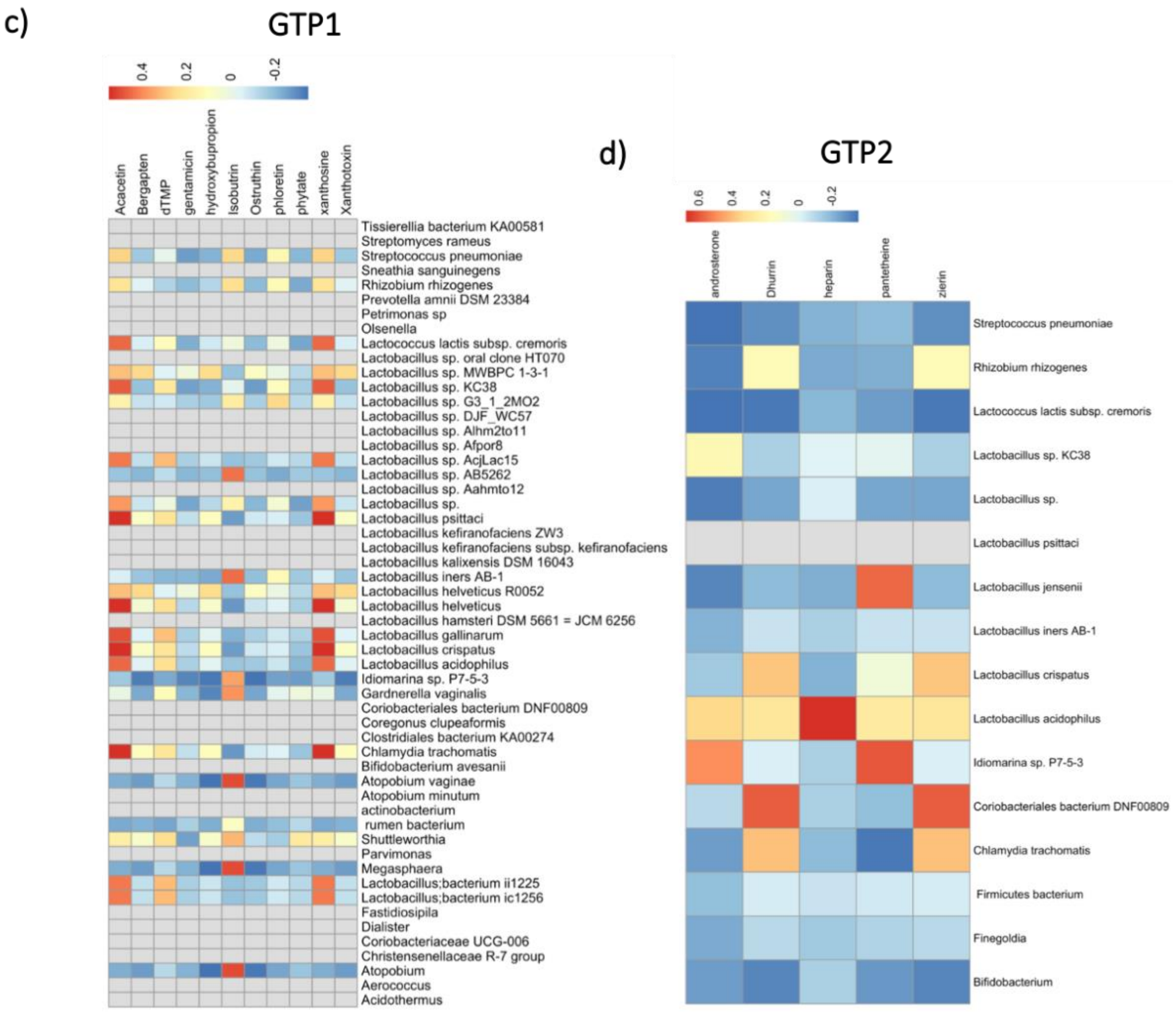

Supplementary Figure 1. Correlations between cervicovaginal fluid metabolites and microbiota according to gestational time points. (**A-B**) community state types (CSTs); (**C-D**) bacterial taxa. GTP1 - 20-22 weeks (n = 13); GTP2 - 26-28 weeks (n = 15). Pearson's correlation coefficients (*r*, colour) range from -1 (dark blue) to +1 (dark red). Only correlations with *P* values < 0.05 are represented. *GTP*, gestational time point.

a)

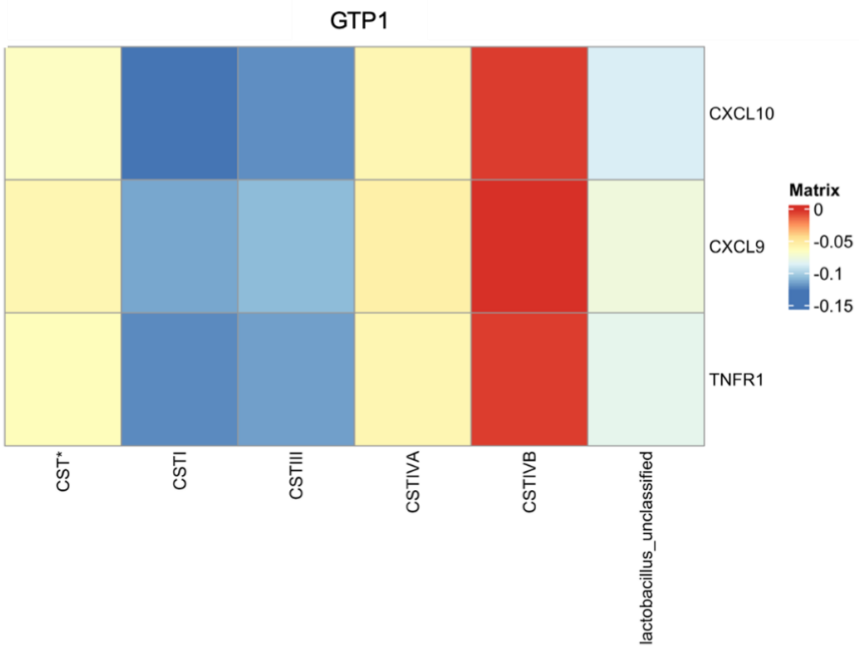
GTP1
CXCL10
CXCL9
TNFR1
Matrix
0
-0.05
-0.1
-0.15
CST*
CSTI
CSTIII
CSTIVA
CSTIVB
lactobacillus_unclassified

b)

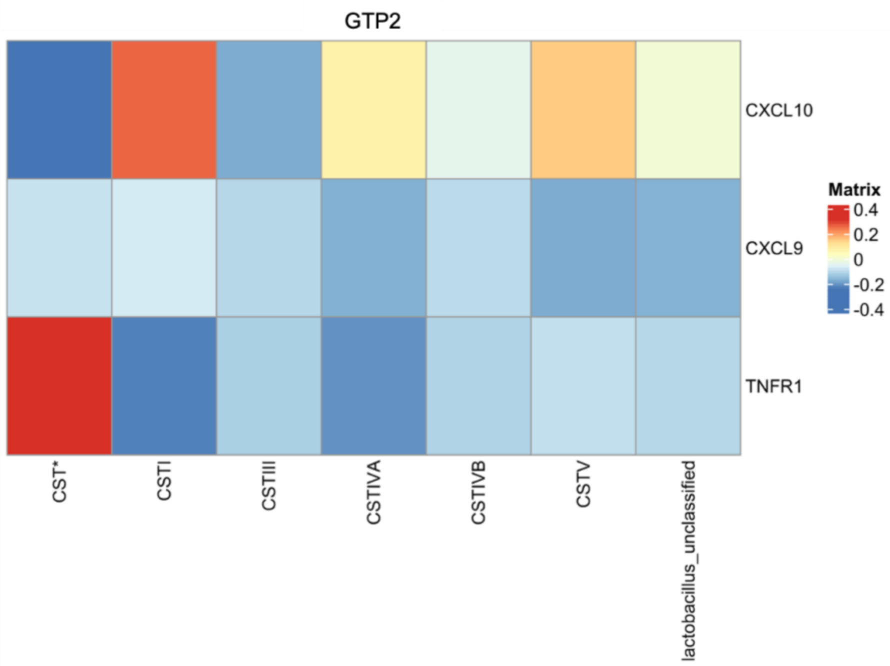
GTP2
CXCL10
CXCL9
TNFR1
Matrix
0.4
0.2
0
-0.2
-0.4
CST*
CSTI
CSTIII
CSTIVA
CSTIVB
CSTV
lactobacillus_unclassified

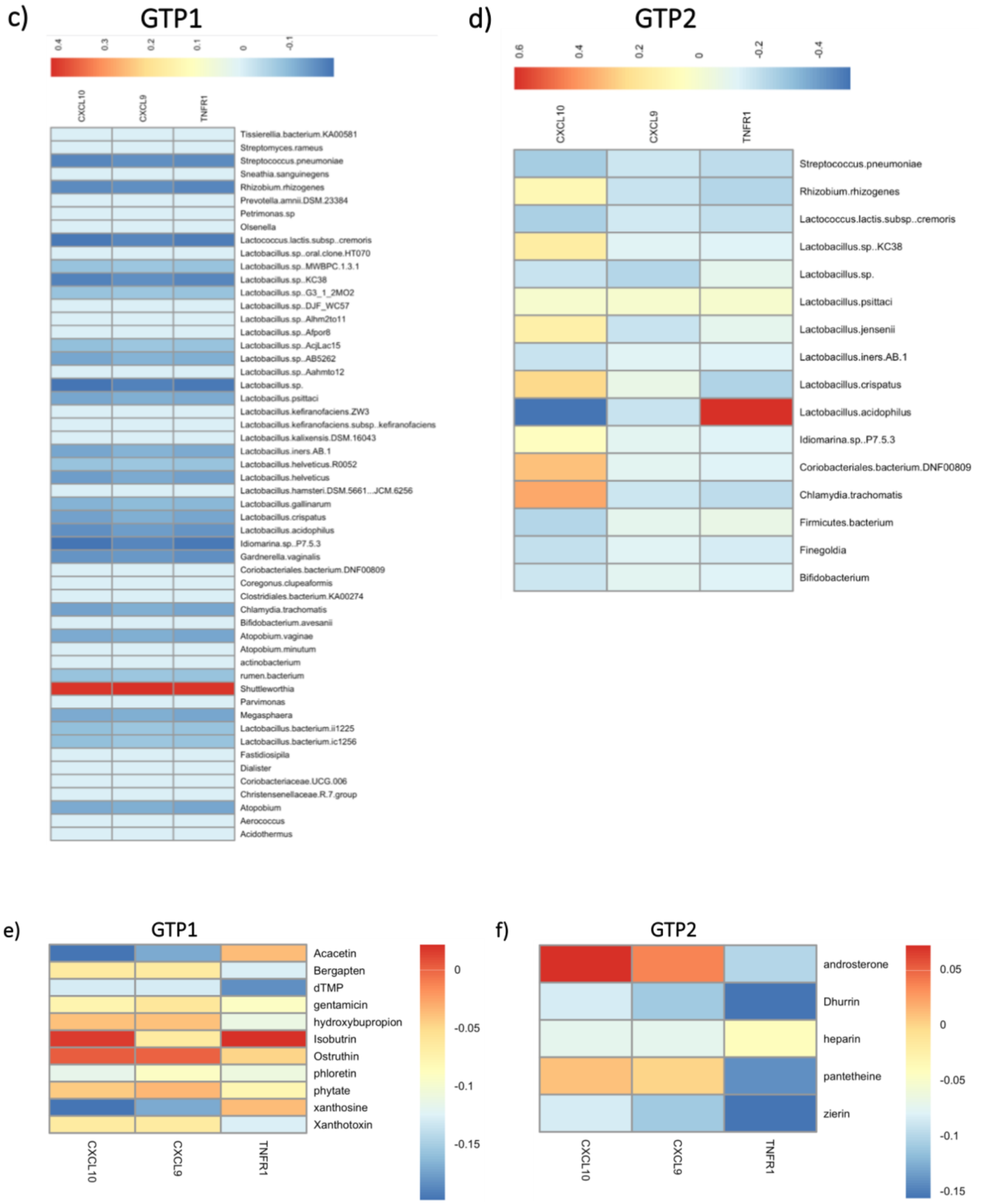

Supplementary Figure 2. Correlations between cervicovaginal fluid inflammatory mediators and microbiota-metabolites. (**A-B**) Community state types; (**C-D**) Bacterial taxa; and (**E-F**) Metabolites. *GTP1:* 20-22 weeks (n = 14); GTP2: 26-28 weeks (n = 14). Pearson's correlation coefficients (*r*, colour) range from -1 (dark blue) to +1 (dark red). Only correlations with *P* values < 0.05 are represented. *GTP*, gestational time point; *CXCL9*, Chemokine (C-X-C motif) ligand 9 or monokine induced by gamma interferon (MIG); *CXCL10*, C-X-C motif chemokine ligand 10 (CXCL10) or Interferon gamma-induced protein 10 (IP10); *DTMP*, deoxythymidine monophosphate; *NAD*, nicotinamide adenine dinucleotide.

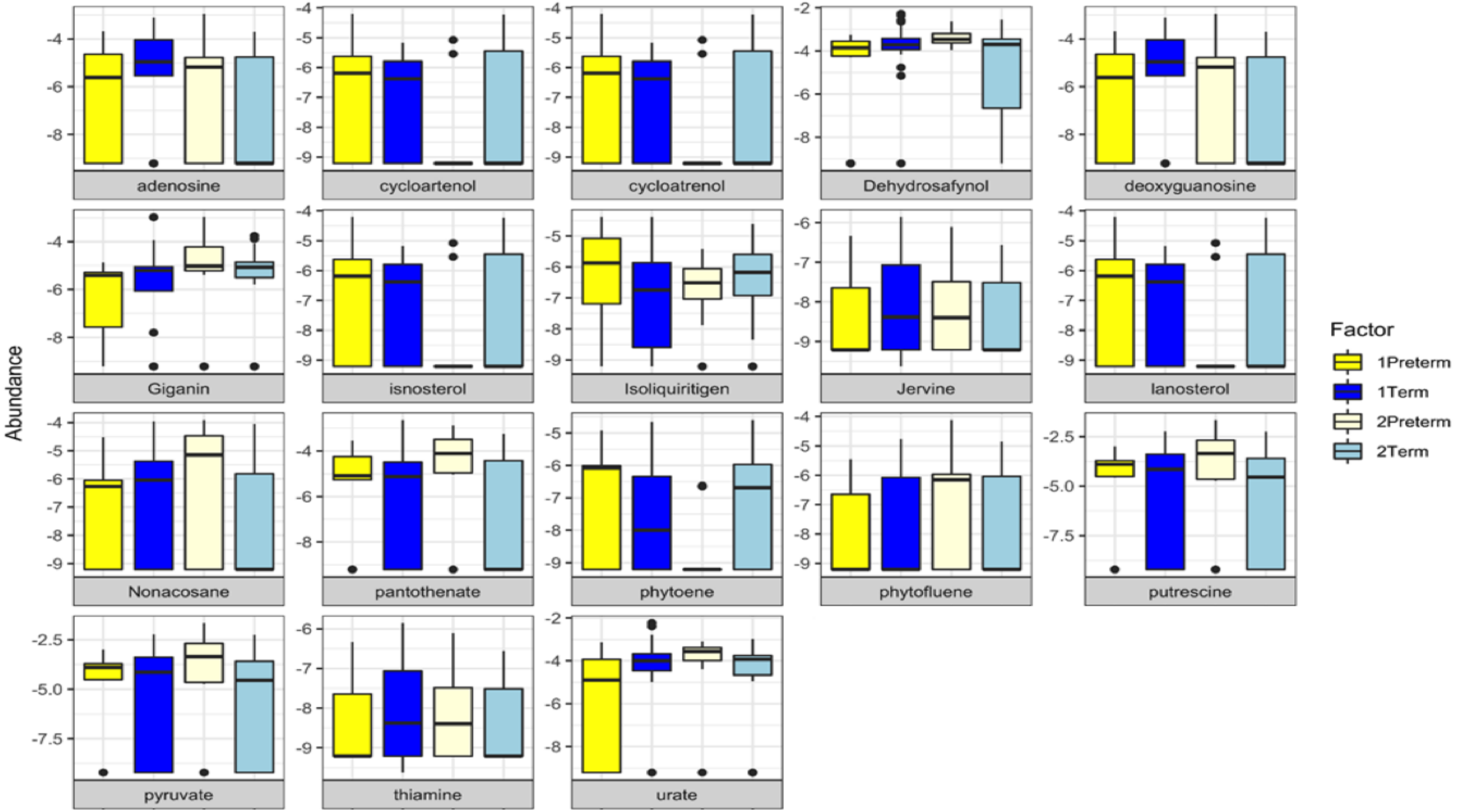

Supplementary Figure 3. Comparison of cervicovaginal metabolite changes across gestational age (GTP1 to GTP2) in preterm-delivered and term-delivered women by ANOVA. GTP1: 20-22 weeks; GTP2: 26-28 weeks; *GTP*, gestational time point.
